# Neuroprogenitor Cells From Patients With TBCK Encephalopathy Suggest Deregulation of Early Secretory Vesicle Transport

**DOI:** 10.3389/fncel.2021.803302

**Published:** 2022-01-13

**Authors:** Danielle de Paula Moreira, Angela May Suzuki, André Luiz Teles e Silva, Elisa Varella-Branco, Maria Cecília Zorél Meneghetti, Gerson Shigeru Kobayashi, Mariana Fogo, Merari de Fátima Ramires Ferrari, Rafaela Regina Cardoso, Naila Cristina Vilaça Lourenço, Karina Griesi-Oliveira, Elaine Cristina Zachi, Débora Romeo Bertola, Karina de Souza Weinmann, Marcelo Andrade de Lima, Helena Bonciani Nader, Andrea Laurato Sertié, Maria Rita Passos-Bueno

**Affiliations:** ^1^Centro de Pesquisas Sobre o Genoma Humano e Células-Tronco, Instituto de Biociências, Universidade de São Paulo, São Paulo, Brazil; ^2^Instituto de Ensino e Pesquisa Albert Einstein, Albert Einstein Hospital, São Paulo, Brazil; ^3^Departamento de Bioquímica, Escola Paulista de Medicina, Universidade Federal de São Paulo, São Paulo, Brazil; ^4^Departamento de Genética e Biologia Evolutiva, Instituto de Biociências, Universidade de São Paulo, São Paulo, Brazil; ^5^Instituto da Criança do Hospital das Clínicas, Faculdade de Medicina da Universidade de São Paulo, São Paulo, Brazil

**Keywords:** STAM, early secretory pathway, vesicle trafficking, autophagy, GM130, clathrin, mTOR, iPSC-neurodevelopmental disease modeling

## Abstract

Biallelic pathogenic variants in TBCK cause encephaloneuropathy, infantile hypotonia with psychomotor retardation, and characteristic facies 3 (IHPRF3). The molecular mechanisms underlying its neuronal phenotype are largely unexplored. In this study, we reported two sisters, who harbored biallelic variants in TBCK and met diagnostic criteria for IHPRF3. We provided evidence that TBCK may play an important role in the early secretory pathway in neuroprogenitor cells (iNPC) differentiated from induced pluripotent stem cells (iPSC). Lack of functional TBCK protein in iNPC is associated with impaired endoplasmic reticulum-to-Golgi vesicle transport and autophagosome biogenesis, as well as altered cell cycle progression and severe impairment in the capacity of migration. Alteration in these processes, which are crucial for neurogenesis, neuronal migration, and cytoarchitecture organization, may represent an important causative mechanism of both neurodevelopmental and neurodegenerative phenotypes observed in IHPRF3. Whether reduced mechanistic target of rapamycin (mTOR) signaling is secondary to impaired TBCK function over other secretory transport regulators still needs further investigation.

## Introduction

Genomic high throughput studies in cohorts of individuals with neurodevelopmental disorders, involving autism spectrum disorder (ASD), epilepsy, and intellectual disability (ID), have recognized several novel rare genetic syndromes. Among them, biallelic pathogenic variants in TBCK (TBC1 domain containing kinase) have been shown to cause infantile hypotonia with psychomotor retardation and characteristic-facies 3 (IHRPF3, OMIM#616900), a severe early onset encephaloneuropathy mainly characterized by developmental delay, hypotonia, and facial dysmorphisms (Bhoj et al., 2016; Chong et al., 2016; Sumathipala et al., 2019). The number of reported cases is still limited to about 40 cases, and the description of novel cases can contribute to a better characterization of the spectrum of clinical variability of this syndrome.

TBCK, a member of the TBC family of proteins, contains a predicted active TBC Rab-GAP domain, which usually functions as GAP for members of the Rab family of small GTPases (also known as small G-proteins), flanked by an N-terminal catalytically inactive kinase domain and a C-terminal rhodanese homology domain (RHOD) (Komurov et al., 2010; Gabernet-Castello et al., 2013). TBCK has been proposed to act on cell proliferation and autophagy through the mechanistic target of rapamycin (mTOR) signaling pathway in non-neural studies (Liu et al., 2013; Ortiz-González et al., 2018). However, to date, less is known about the mechanism of action of TBCK, being unknown whether disruption of mTOR signaling is a primary or secondary effect of loss of TBCK. In addition, depending on the cell type, TBCK has been associated with both tumor-promoting and tumor-suppressive function (Liu et al., 2013; Wu et al., 2014; Wu and Lu, 2021), suggesting variable regulation according to the cell type. Although biallelic loss-of-function variants in TBCK are associated with several neural clinical phenotypes, functional studies of TBCK in neural cells are still lacking.

In this study, we described two new IHPRF3-affected sibs that present autism as an additional clinical feature of the syndrome and are compound heterozygotes for loss-of-function variants in TBCK. In order to gain more insights into mechanisms of action of TBCK in neural cells and to better understand the pathophysiology of IHPRF3 syndrome, we employed neuroprogenitor cells (iNPC) derived from induced pluripotent stem cells (iPSC) from the two IHPRF3-affected sibs. Colocalization analysis of the TBCK protein with early secretory pathway markers suggests altered ER-to-Golgi vesicle transport as a possible mechanism for the impaired autophagosome biogenesis, cell cycle progression, and migration observed in cells of patients. iNPC of patients also showed decreased mTOR signaling, which may be secondary to impaired regulation of TBCK-mutated protein over other early secretory vesicle transport regulators.

## Materials and Methods

### Subjects

The two affected sisters (F6331-1 and F6331-4) were ascertained after initial diagnosis of ASD and the following referral to the genetic counseling service at Centro de Estudos do Genoma Humano e Células-Tronco (CEGH-CEL), Universidade of São Paulo (USP). Patients were routinely diagnosed based on DSM-5 (American Psychiatric Association, 2013) and CARS evaluation.

Control individuals (F7007-1, F8799-1, and F10006-1; one male and two females, respectively, aged 31, 29, and 33 years) are healthy and unrelated to the affected individuals.

This study was approved by the National Ethics Committee (Comissão Nacional de Ética em Pesquisa no Brazil, Process no. CAAE43559314.0.0000.5464). Blood from all the individuals was collected after a signed written informed consent by the participants of the study or their legal representatives.

### Investigation of Pathogenic Variants

#### DNA Extraction

Genomic DNA extraction from whole blood was performed using the QIAsymphony automated workstation, following the instructions of the manufacturer (Qiagen, United States).

#### Selection of Whole-Exome Sequencing and Variants

Whole-exome sequencing (WES) and bioinformatic analysis were performed at the CEGH-CEL sequencing facility. Exome capture was carried out using the TruSeq Exome Library Prep Kit (Illumina, Inc., United States) following the recommendations of the manufacturer. HiSeq 2500 sequencer (Illumina) was used for sequencing paired-end reads of approximately 100 bp × 100 bp. Reads were aligned with Burrows-Wheeler Aligner (BWA), against the hg19 reference genome (Li and Durbin, 2009). Data processing and variant calling were carried out on Picard and Genome Analysis Tool Kit (GATK) (McKenna et al., 2010). ANNOVAR (Wang et al., 2010) was used to annotate variants. Candidate pathogenic variants were filtered according to the following criteria: (a) exclusion of low-quality variants; (b) inclusion of rare variants with minor allele frequency (MAF) < 0.01 in reference databases (i.e., 1,000 Genomes Project (1000G), National Institutes of Health; 6,500 Exome Sequencing Project (6500ESP), Washington University; and ABraOM, University of São Paulo (Naslavsky et al., 2017); (c) inclusion of variants with frequency < 0.05 in internal control samples (i.e., DNA samples that were sequenced and processed in the same batch); (d) exclusion of polymorphic genes (Fajardo et al., 2012); and (e) exclusion of variants located within the last three amino acids of a protein. We prioritized homozygous or compound-heterozygous loss-of-function (LoF) variants shared between the two affected siblings. The position of the candidate LoF variant identified was manually converted to GRCh38/hg38, using the UCSC website^1^.

#### Sanger Sequencing

Sanger sequencing of genomic DNA and cDNA was performed for the validation of the candidate variant and segregation analysis. Primers for PCR amplification and sequencing were designed on Primer-BLAST (NCBI;^2^) (Supplementary Table 1).

#### Array Comparative Genomic Hybridization and Real-Time Quantitative PCR for Copy Number Analysis

Array comparative genomic hybridization (aCGH, 180K, Agilent, United States) was performed in both patients according to the recommendations of the manufacturer. The analysis was carried out using Agilent Genomic Workbench 7.0 (Agilent Technologies, Santa Clara, CA, United States).

Real-time quantitative PCR (RT-qPCR) of TBCK was performed to detect the predicted small deletion in the region chr4:107,071,580−107,113,380. Six primer pairs were designed on Primer-BLAST (Supplementary Table 2). Relative quantification was carried out by normalization to GAPDH, and quantification data were calibrated relative to a control without any known CNV in TBCK (D’haene et al., 2010).

### iPSC Reprogramming

Induced pluripotent stem cells were reprogrammed from peripheral blood mononuclear cells (PBMC), using a non-viral method with non-integrating plasmids (Okita et al., 2013), which was established at HUG-CELL with minor modifications (Griesi-Oliveira et al., 2015; Miller et al., 2017). AMAXA nucleoporator (Lonza, Basel, Switzerland) was used for the transfection of plasmids containing the transcription factors OCT-4, SOX2, KLF4, L-MYC, and LIN28. Each reprogrammed cell line was cocultivated with irradiated murine embryonic fibroblasts (MEF, Millipore) in DMEM/F12 medium supplemented with 2mM GlutaMAX-I, 0.1 mM non-essential amino acids, 55 μM 2-mercaptoethanol, 30 ng/ml fibroblast growth factor (FGF-2), and 20% of knockout serum replacement (KSR, Life Technologies). iPSC colonies with typical morphology were then transferred to Matrigel (BD-Bioscience) coated plates and fed with E8 medium (Life Technologies). All iPSC colonies were tested for plasmid integration into the host genome and excluded for the presence of aneuploidies with SALSA MLPA P070 Human Telomere-5 probe mix (MRC-Holland). Cell pluripotency was evaluated through stem cell markers prior to differentiation to iNPC.

### Neuroprogenitor Cells Differentiation

Differentiation of iPSC into iNPC was carried out using a protocol previously established at HUG-CELL (Griesi-Oliveira et al., 2015). In brief, iPSC were cultivated in 0.5 × NB medium (1/2 DMEM/F12: 1/2 Neurobasal media with 0.5 × N-2 supplement (100×) (Thermo Fisher Scientific) and 0.5 × B27 minus vitamin A supplement (50×) (Thermo Fisher Scientific) supplemented with Dorsomorphin, 1 μM (Tocris) for 2 days. Then, iPSC were harvested with accutase treatment, and cell clumps were manually transferred into low-attachment plates (Corning) on 0.5 × NB medium supplemented with 1 μM dorsomorphin. The next day, the medium was replaced with 0.5 × NB medium supplemented with 20 ng/ml FGF-2 and 20 ng/ml epidermal growth factor (EGF) (Thermo Fisher Scientific) and allowed to grow in suspension for 7 days. Then, the resulting embryoid bodies were lightly dissociated with accutase and plated on matrigel-coated plates, from which rosettes start to form within 4–7 days. The selected rosettes were manually collected and plated in poly-L-ornithine (10 μg/ml; Sigma) and natural mouse laminin (5 μg/ml; Invitrogen) coated plates for iNPC expansion. All iNPC were characterized through neural progenitor markers.

Except in cell cycle and BrdU incorporation assays, for which we used iNPC only from patient F6331-1, in each experiment performed in this study, we used iNPC from all individuals (patients: F6331-1 and F6331-4; and controls F7007-1, F8799-1, and F10006-1).

### Neurosphere Formation (3D Model)

Notably, 25 μl drops of cell suspension containing a total of 4 × 104 iNPC (*N* = 5, in passage number ranging between 6 and 8) in 0.5 × NB medium containing 20 ng/ml FGF-2 and 20 ng/ml EGF were placed onto the lid of a 10-cm dish and set to rest for 48 h at 37°C and 5% CO_2_. After this period, the cells aggregated into homogeneous neurosphere sizes were placed into 6-well low attachment plates in 0.5 × NB medium supplemented with 20 ng/ml FGF-2 and 20 ng/ml EGF.

### Cell Culture Conditions

mTOR signaling activity and autophagy in iNPC were evaluated in normal culture condition and in the following three stress-inducing conditions: (i) absence of EGF and FGF-2 for 24 h; (ii) absence of EGF and FGF-2 and concomitant incubation with mTOR inhibitor, rapamycin (100 nM) for 24 h; and (iii) absence of EGF and FGF-2 for 24 h and subsequent addition of brefeldin A (BFA, 4 μM), which induce Golgi complex fragmentation, for 3 h.

### RNA Extraction and RT-qPCR

Total RNA from all iPSC and iNPC cultures was isolated using NucleoSpin RNA II Kit (Macherey-Nagel, Thermo Fisher; Merk Millipore), and cDNA was synthesized by reverse transcription using SuperScript IV (Thermo Fisher Scientific).

Primer pairs for *TBCK*, *CLTC*, *CLTD*, *RAB5A*, *STAM1*, and *STAM2* (Supplementary Table 3) were designed at Primer-BLAST, while primers of pluripotency markers (*OCT3/4* and *NANOG*), neural markers (*PAX6* and *SOX1*), and housekeeping genes (*GAPDH* and *TBP*) were adopted from the literature (Ishiy et al., 2015; Miller et al., 2017). Each sample was analyzed in triplicate with the use of Fast SYBR Green PCR Master Mix (Applied Biosystems) according to the recommendations of the manufacturer. The reactions were run in a QuantStudio^®^ 5 Real-Time PCR system (Applied Biosystems). The expression of each gene was normalized to GAPDH or TBP housekeeping gene, and the results are shown as the mean fold change of the normalized gene expression relative to a calibrator sample. For pluripotency or neural markers, another cell type with no expression of the gene tested was used as a negative control.

### Cell Lysis and Western Blot

Cells were homogenized in RIPA buffer (Thermo Fisher Scientific) containing protease and phosphatase inhibitor cocktails (Sigma). Lysates were incubated on ice for 10 min and then centrifuged at 8,000 × *g* for 15 min at 4°C, and the supernatants of total cell lysates were collected. Protein concentrations were determined with a BCA microprotein assay kit (BioAgency). A total of 20 μg of protein from each sample were separated by sodium dodecyl sulfate-polyacrylamide gel electrophoresis (SDS-PAGE) and transferred to nitrocellulose membranes, which were blocked with 5% bovine serum albumin (BSA) for 1 h and then incubated with primary antibodies overnight at 4°C. Detection was performed using horseradish peroxidase-coupled anti-mouse or anti-rabbit secondary antibodies (1:2,000, Cell Signaling Technology), enhanced chemiluminescence (ECL) substrate (GE Healthcare), and ImageQuant LAS-4000 (GE Healthcare). The intensity of the bands was determined by densitometry using NIH ImageJ software (Bethesda, MD, United States)^3^. The following primary antibodies were used: anti-TBCK (1:50, #sc-81865, Santa Cruz Biotechnology), anti-pRPS6240/244 (1:5,000, #5364), anti-LC3A/B (1:1,000, #12741), anti-BECN1 (1:1,000, #3738), anti-p62/SQSTM1 (1:1,000, #8025) from Cell Signaling Technology, anti-Cathepsin D (1:2,000, #ab75852), anti-Vinculin (1:5,000, #ab18058) from Abcam, and anti-βactin (1:15,000, A2228 Sigma) antibodies. All target protein levels were quantified and normalized to the corresponding β-actin or vinculin levels.

### AKT/Mechanistic Target of Rapamycin Multiplex Assay

mTOR signaling activity from iNPC cultured in the absence of EGF and FGF-2 for 24 h was also assayed using the MILLIPLEX MAP^®^ Akt/mTOR Phosphoprotein Magnetic Bead 11-Plex panel (#48-611MAG) and the MILLIPLEX MAP AKT/mTOR 11-plex panel (#48-612MAG) (Millipore) with 10 μg of total protein extracts of each sample, according to the instructions of the manufacturer.

### Immunofluorescence

iPSC and iNPC grown on coverslips were fixed in 4% Paraformaldehyde (PFA), permeabilized in 0.2% Triton X-100 in Phosphate Buffered Saline (PBS) for 30 min at 4°C, and blocked for 1 h in PBS containing 5% BSA, and then incubated overnight at 4°C with primary antibodies diluted in the same blocking buffer. Primary antibodies used are as follows: anti-SOX1 (1:200, #4194S), anti-RAB7 (1:200, #9367), anti-LC3 (1:200, #3868) from Cell Signaling Technology; anti-SOX2 (1:100, #ab171380) and anti-GM130 (1:50, #ab52649) from Abcam; anti-COPII (1:200, #PA1-069A) from Thermo Fisher Scientific; anti-TBCK (1:50, #sc-81865), anti-RAB5A (1:100, #sc-309), anti-clathrin (1:200, #sc-6579), and anti-caveolin (1:200, #sc-894) from Santa Cruz Biotechnology; anti-STAM (1:200, #12434-1-AP, Proteintech). Cells for anti-LC3 labeling were fixed with methanol and proceeded with the same protocol described. Then, cells were washed 3 times with PBS and incubated with secondary antibodies conjugated with Alexa fluor 488 or Alexa fluor 680 for 1 h at room temperature. After another washing step, the cells were mounted in a Vectashield mounting medium with 4′,6-diamidino-2-phenylindole (DAPI) (1 μg/ml, Vector Labs). Samples were analyzed using a confocal laser scanning microscope, LSM800 (Zeiss), using an immersion lens (63 × /1.40 Oil). Each channel was imaged separately. The acquired fluorescent images were converted to tiff format and further processed using Fiji software. Background from all the images was subtracted using the Fiji background subtraction algorithm (rolling ball radius = 50 pixels). To quantify TBCK colocalization with Caveolin, Clathrin, RAB5A, STAM, GM130/GOLGA2, and COPII, the Manders method of correlation (MCC) was performed using ImageJ/Fiji-Coloc2 plugin. For the analysis of LC3 puncta, the binary images generated after background subtraction were filtered for particles with Feret diameter larger than 100 nm. The number of puncta was calculated by dividing the total number per number of cell nuclei labeled in a given image. Then, we calculated the mean and SD for the puncta number and puncta size (Kjos et al., 2017) of each sample.

### Cell Cycle and Cell Proliferation Analysis

A total of 5 × 105 iNPC from each sample (in similar passage number, ranging between 5 and 6) were seeded in duplicate into 6-well plates (Corning) in 0.5 × NB medium supplemented with 20 ng/ml EGF and 20 ng/ml FGF-2. The day after seeding, EGF and FGF-2 were removed from 0.5 × NB medium during 24 h for cell cycle synchronization at G0/G1 (T0) (Mazemondet et al., 2011). After this period, the medium was replaced with 0.5 × NB medium supplemented with EGF and FGF-2 to induce cell cycle progression and cell proliferation. Cells were collected at T0 (right after 24 h of EGF and FGF-2 deprivation), 24 and 40 h after growth factor supplementation (T24 and T40, respectively).

Three hours before collection of the cells, at each time point, 20 μM BrdU was added (Sigma-Aldrich). Then, iNPC were rinsed twice with PBS, harvested with trypsin incubation, and fixed in 70% EtOH overnight at −20°C. After complete fixation, cells were double-stained with propidium iodide (PI) and anti-BrdU (BrdU Monoclonal Antibody, FITC, Invitrogen), in order to ascertain the cell distribution through cell cycle stages (G0/G1, S, and G2/M) and cell proliferation, respectively. Appropriate assay controls were used in the assay (unstained sample; PI-stained- and anti-BrdU-stained-only samples), and at least 5,000 events were acquired. Data were analyzed using Guava Express PRO software (Millipore) and gated to remove debris and cell clumps.

### Apoptosis Analysis

A total of 2 × 105 iNPC were seeded into a 12-well plate (Corning), in duplicate. In the following day, cells were incubated with 20 μl of CellEvent^®^ Caspase-3/7 Green ReadyProbes Reagent (Thermo Fisher Scientific) for 1 h, at 37°C, and 5% CO_2_. Then, iNPC were rinsed with PBS, fixed with 4% PFA, and the nuclei stained with Vectashield mounting medium with DAPI. Images were captured using a Nikon fluorescence microscope (Nikon Eclipse Ti-E, Nikon). The quantification of caspase marked cells was performed using ImageJ software from three different fields from each duplicate. We determined the total number of cells by DAPI nuclear staining.

### Cell Migration

For radial migration assay, 25 μl drops of cell suspension containing a total of 5 × 104 iNPC (in passage number ranging between 5 and 6) in 0.5 × NB medium containing 20 ng/ml FGF-2 and 20 ng/ml EGF were placed onto the lid of a 10-cm dish and set to rest for 48 h at 37°C and 5% CO_2_. After this period, the cells aggregate into homogeneous neurosphere size. A total of 4 neurospheres per sample were then transferred to poly-L-ornithine (10 μg/ml; Sigma) and natural mouse laminin (5 μg/ml; Invitrogen) coated plates in 0.5 × NB medium containing 20 ng/ml FGF-2 and 20 ng/ml EGF. Images were captured at 4, 8, and 24 h after adhesion to the coated plates. The pictures were acquired using the EVOS Cell Imaging System (Thermo Fisher Scientific). Cell migration was analyzed using ImageJ software. For each time point, we measured the neurosphere outer edges. Cell migration was estimated by the outer diameter of neurosphere migration normalized to inner neurosphere diameter (μm) (Ge et al., 2016).

### Statistical Analysis

Data were analyzed using the non-parametric unpaired two-tailed Wilcoxon-Mann-Whitney test or one-way ANOVA followed by the recommended correction test, using Graphpad 7.0 (Graphpad Software Inc., La Jolla, CA, United States). A regression *via* decision tree was used to analyze cell-cycle progression, using the R package (R Core team, 2019). For functional enrichment analysis of proteomic data, Fisher’s exact test was applied. *p*-values < 0.05 were considered statistically significant. In the figures, statistical significances are indicated as follows: *p < 0.05, **p < 0.01, and ***p < 0.001. Data are expressed as mean ± SD.

## Results

### Clinical Presentation of Patients

**FIGURE 1:**
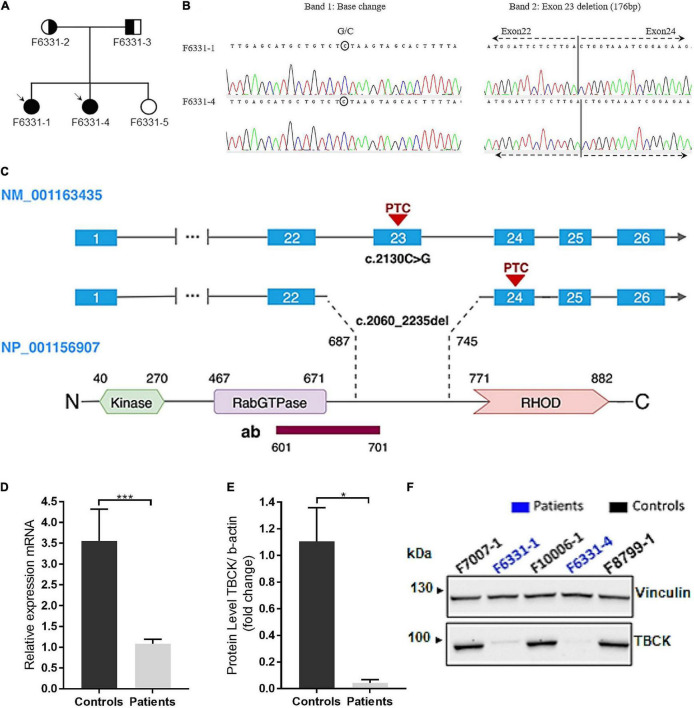
IHPRF3 patients harboring biallelic pathogenic variants in TBCK and relative quantification of mRNA and protein expression levels in iNPC. **(A)** Pedigree of family F6331. Circle—female; square—male; symbol-filled—affected patients; symbol empty—not affected sister; circle half-filled—harbors the heterozygous microdeletion; square half-filled—harbors the heterozygous stopgain variant. **(B)** Chromatograms of the cDNA sequence show that both alleles are expressed: in the left image, a base change in Band 1 (expected base G changed to a C in reverse strand) represents the stopgain variant; and in the right image, the juxtaposition of exons 22 and 24 (excision of exon 23) in Band 2 representing the allele with the microdeletion. Bands 1 and 2 represent cDNA PCR products for TBCK in agarose gel (Supplementary Figure 2A). **(C)** Schematic representation of TBCK transcript and protein. The illustration shows the two mutant isoforms identified in the patients and the predicted positions on the protein. The antibody target region is also represented. The pathogenic variants create a putative premature termination codon (PTC) in exons 23 and 24, located within the C-terminal region of the protein, upstream of the RHOD domain. **(D)** TBCK mRNA expression (data from TBCK-mRNA-3 primer pair) shows reduced transcript levels in patients, compared with controls. Each biological sample had three technical replicates, and each individual was considered a biological replicate. **(E,F)** Western blot (WB) shows low levels of TBCK protein in iNPC of patients, compared with iNPC of controls, which are compatible with an autosomal recessive inheritance condition. *p-value < 0.05, ***p-value < 0.001, and n.s.—not significant. Data on graphs are shown as mean ± SD of technical replicates and biological samples. Patients: F6331-1 and F6331-4 (*N* = 2); controls: F7007-1, F8799-1, and F10006-1 (*N* = 4).

The two affected sisters in this study reported were born from a non-consanguineous healthy couple (Figure 1A) at 38 weeks of gestation *via* vaginal delivery, with normal anthropometric measurements (Table 1). The proband (Patient F6331-1), first evaluated at CEGH-CEL by the age of 6 years, presented with global developmental delay. At the age of 3 months, her parents noticed she had low muscle tone (e.g., she could not hold her neck and head). Later, they noticed delayed unassisted sitting (after 12 months) and walking (after 30 months), as well as delayed speech (first words after 24 months), which was limited to a few words. At the age of 3 years, she was diagnosed with autism and ID, and at the age of 7 years, she had a severe psychotic episode. Further clinical evaluation showed several dysmorphic features, prominent digit pads, metabolic abnormalities like dyslipidemia and hypothyroidism, and brain MRI at 12 years showed white matter volumetric reduction and T2 hyperintensity (Table 1 and Supplementary Figure 1A). ECG and EEG were normal. At 14 years of age, she attended a special school and only said a few words and short sentences and showed inattention and lack of motivation to engage in educational activities.

**TABLE 1:** Clinical characteristics of two patients with IHPRF3.

Patient	F6331-1	F6331-4
Age (year), Sex	14, F	10, F
**Birth evaluation**		
°Birth Weight (centile)	3,990 g (p90)	3,850 g (p50)
°Birth Length (centile)	51 cm (p75)	48 cm (p50)
°Apgar Scores (1st/5th min)	10/10	8/9
**Last evaluation**		
°Weight (centile)	53.5 kg (p90)	33.5 kg (p75)
°Height (centile)	164 cm (*p* > 97)	133 cm (p50)
°Head circumference (centile)	55 cm (p96)	53.5 cm (*p* < 96)
**Development**		
°Regression	No	yes
°Seizures	no	yes
°Speech	Few words	Non-verbal
°Neuropsychomotor development	Severe delay	Severe delay
°Cognitive	Severe delay	Severe delay
°ASD	yes	yes
°ID	yes	yes
°Hypotonia	yes	no
°Motor	Motor delay	Motor dyspraxia
EEG	No	Diffuse bilateral slow waves with multifocal epileptiform discharges
MRI	Volumetric reduction and abnormal T2 signal hyperintensity in white matter	Volumetric reduction and abnormal T2 signal hyperintensity in white matter
Facial dismorphism	Deep-set eyes, prominent nasal bridge, accentuated Cupid’s bow of the upper lip, high palate, widely spaced teeth; bitemporal narrowing	Deep-set eyes, prominent nasal bridge, accentuated Cupid’s bow of the upper lip, high palate, widely spaced teeth, bitemporal narrowing
Other features	Prominent digit pads	prominent digit pads, brachydactyly of the 4th and 5th toes and hypoplastic nails
Metabolic alterations	dyslipdemia and hypothyroidism	dyslipdemia and hypothyroidism

*ASD, autism spectrum disorder; ID, intellectual disability; EEG, electroencephalogram; MRI, magnetic resonance imaging.*

The second affected sister (Patient F6331-4), first evaluated at CEGH-CEL by the age of 3 years, showed a better initial psychomotor developmental course. She sat unaided at about 6 months and could walk unassisted at about 14 months, but at around 12 months, she was diagnosed with autism and global developmental delay. She had the first seizure episode at the age of 4 years and was diagnosed with focal and generalized epilepsy at approximately 6 years of age. EEG demonstrated diffuse bilateral slow waves with multifocal epileptiform discharges. Clinical evaluation at the age of 7 years showed similar dysmorphic features to her sister F6331-1, in addition to brachydactyly of the fourth and fifth toes and hypoplastic nails. She also developed metabolic abnormalities, and an MRI of the brain at the age of 9 years showed a volumetric reduction of the white matter (Supplementary Figure 1B). At 10 years of age, she showed progressive weakness in her lower limbs, first noticed approximately at the age of 8 years. ECG was normal.

### Biallelic Loss-of-Function Variants in TBCK Cosegregate With the Syndrome

To investigate the genomic cause of the clinical phenotypes of patients, we performed array comparative genomic hybridization (aCGH), whole-exome sequencing (WES), and subsequent site-specific quantification of genomic DNA using the RT-qPCR. WES and RT-qPCR analyses revealed that the sisters share rare LoF variants in exon 23 of the TBCK gene: a stopgain variant (NM_001163435: c.2130C > G; p.Tyr710*) and a microdeletion (NM_001163435: c.2060_2235del; p.Glu687Valfs9*) (Figures 1B,C and Supplementary Figure 2). Conventional PCR and Sanger sequencing, using either genomic DNA or cDNA isolated from iNPC validated both variants and are better described below.

cDNA sequencing revealed that the microdeletion leads to the loss of exon 23 and juxtaposition of exons 22 and 24, resulting in a predicted premature termination codon (PTC) in exon 24 (Figures 1B,C and Supplementary Figure 2C). Segregation analysis showed that the stopgain variant was inherited from their father and the microdeletion from their mother (Supplementary Figures 2A,B), while the unaffected sib did not inherit any of these variants. Both variants were considered pathogenic according to the American College of Medical Genetics and Genomics (ACMG) guidelines (Richards et al., 2015). No additional disrupting variants following an autosomal recessive inheritance model were identified by WES or aCGH.

Quantitative analyses showed that iNPC of patients exhibited over the twofold decrease in *TBCK* mRNA expression, both truncated alleles were transcribed (Figures 1B,D), and through Western blot (WB), we observed a single faint band with the same electrophoretic mobility as the wild type (WT) *TBCK* protein in controls (Figures 1E,F). These results show that the C-terminal variants identified in these patients cause a strong disruption in TBCK translation.

### Patient-Derived Neuroprogenitor Cells Under Stress-Inducing Conditions Exhibit Downregulation of Mechanistic Target of Rapamycin Signaling Activity

To explore the neural functional consequences of the identified variants in *TBCK*, we differentiated iNPC from iPSC from the 2 IHPRF3 patients and 3 unrelated controls. All iPSC colonies were positive for pluripotency markers (*OCT3/4* and *NANOG*) (Supplementary Figures 3A,B), and the iNPC were positive for neural stem cell markers (*SOX1*, *SOX2*, and *PAX6*) (Supplementary Figures 3C,E), suggesting successful cell reprogramming and differentiation.

**FIGURE 2:**
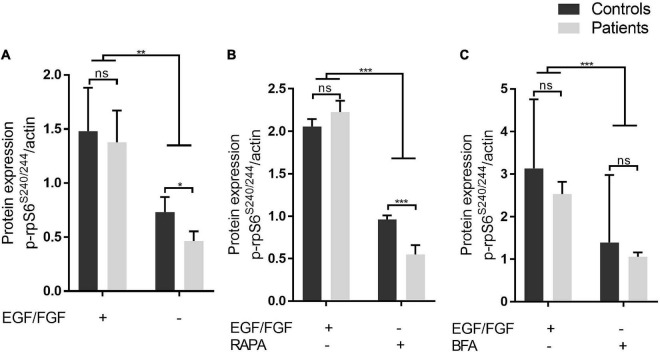
Under stress-inducing conditions, patient-derived iNPC exhibits reduced RPS6 phosphorylation. **(A–C)** Protein level of phospho-RPS6 (residues S240-244) from iNPC cultivated under normal growth conditions and three stress-inducing conditions. iNPC without epidermal growth factor (EGF) and FGF-2 for 24 h only **(A)**, without EGF/FGF-2 and concomitant treatment with rapamycin (100 nM) for 24 h **(B)**, or without EGF/FGF-2 and subsequent treatment with brefeldin A (BFA, 4 μM) for 3 h **(C)**. *p-value < 0.05. WB experiments were replicated twice. Data are shown as mean ± SD of technical replicates and biological samples. Patients: F6331-1 and F6331-4 (*N* = 2); controls: F7007-1, F8799-1, and F10006-1 (*N* = 3). **p-value < 0.01, ***p-value < 0.001, ns - not significant.

First, to investigate whether iNPC from IHPRF3 patients show abnormal mTOR signaling activity, we analyzed by Western blot the phosphorylation levels of the ribosomal protein RPS6, a downstream target of the mTORC1 signaling pathway. Phosphorylated RPS6 (p-RPS6S240/244) was quantified in iNPC grown in normal conditions (in the presence of EGF and FGF-2 growth factors) and in three stress-inducing conditions. Under normal growth conditions, we did not detect any difference in p-RPS6S240/244 levels between patient-derived and control-derived iNPC (Figures 2A,C and Supplementary Figures 4A,C,D). Intracellular stress caused by the withdrawal of EGF/FGF-2 alone significantly decreased RPS6 activation in iNPC of patients in comparison to controls (p-RPS6S240/244 protein level: patients—0.55 ± 0.11 vs. controls—0.96 ± 0.05; Mann–Whitney test, two-tailed, *p*-value = 0.0238), and the same was observed under EGF/FGF-2 withdrawal combined with treatment with mTORC1 complex inhibitor rapamycin (p-RPS6S240/244 protein level: patients—0.46 ± 0.09 vs. controls—0.73 ± 0.13; Mann–Whitney test, two-tailed, *p*-value = 0.0286) (Figures 2A,B and Supplementary Figures 4B,C). Conversely, inhibition of mTOR signaling through EGF/FGF-2 deprivation for 24 h and subsequent addition of BFA (which interferes with glutamine-induced lysosomal localization and activation of mTOR—Jewell et al., 2015; Meng et al., 2020) led to similar RPS6 phosphorylation in both groups (Figure 2C and Supplementary Figure 4D). Together, these results show that mTOR signaling in patient-derived iNPC is altered only under specific stress-inducing conditions and suggest that TBCK acts through an indirect mechanism over mTOR signaling.

### TBCK Colocalizes With Proteins Involved in Both Endocytic and Secretory Pathways

**FIGURE 3:**
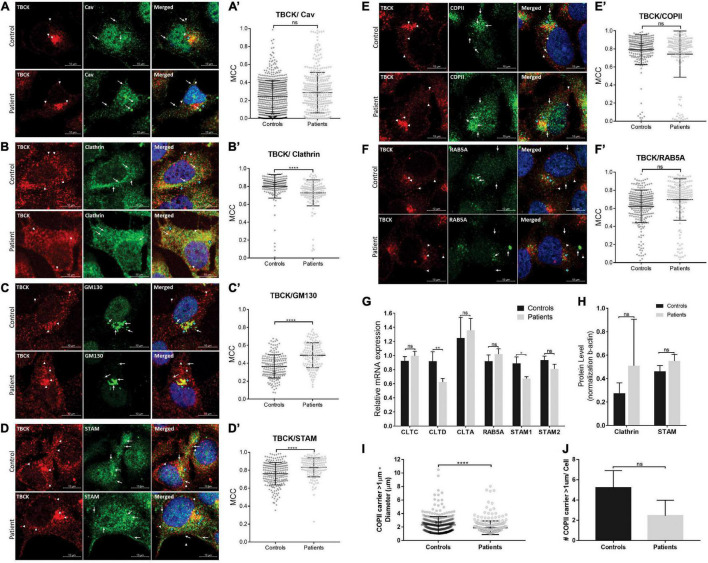
Patient-derived iNPC show altered colocalization levels with endocytic and early secretory pathway markers. **(A–F)** Representative images of confocal microscopy of iNPC coimmunostained for endogenous TBCK and specific vesicle transport machinery components: **(A)** Caveolin (Cav), **(B)** Clathrin, **(C)** GM130/GOLGA2, **(D)** STAM, **(E)** COPII, and **(F)** RAB5A. For more detailed images, see Supplementary Figure 5. White arrowheads indicate TBCK dots; white arrows indicate the vesicle transport regulators indicated on each image; blue arrows indicate the colocalized dots. **(A′–F′)** Graphs of Manders coefficient colocalization (MCC) of TBCK with each target protein. Measurements were performed using ImageJ/Fiji-Coloc2 plugin. Error bars indicate SD (each dot represents the MCC in each cell; data represent the analysis of *n* > 150 cells/individual). **(G)** Relative mRNA expression of vesicle transport regulators in iNPC grown as neurospheres show reduced clathrin heavy chain CLTD and STAM1 transcript levels in neurospheres of patients, compared with neurospheres of controls. **(H)** Clathrin and STAM immunoblot densitometries reveal a tendency to increase protein levels in neurospheres of patients, compared with neurospheres of controls. **(I,J)** Analysis of large COPII carrier (>1 μm) average diameter **(I)** and number per cell **(J)**. ****p-value < 0.0001. Unpaired *t*-test, two-tailed. Data are shown as mean ± SD of biological replicates. 4′,6-diamidino-2-phenylindole (DAPI) (blue) marks cell nuclei. Scale bar = 10 μM. Patients: F6331-1 and F6331-4 (*N* = 2); controls: F7007-1, F8799-1, and F10006-1 (*N* = 3). *p-value < 0.05, **p-value < 0.01, ns - not significant.

To evaluate whether *TBCK* plays a role in intracellular vesicle transport in iNPC, we first investigated intracellular localization of TBCK through the analysis of immunocolocalization levels with several proteins involved in endocytic (caveolin, RAB5A) and/or secretory (COPII, GM130/GOLGA2) pathways, in addition to Clathrin and STAM implicated in both. In iNPC of patients and controls growing under normal culture conditions, we observed that TBCK protein was diffusely distributed in the cytosol, together with a more condensed perinuclear localization, suggesting that a fraction of TBCK-mutated protein is translated and that this protein is not retained in any particular cellular compartment in iNPC of patients (Supplementary Figures 5A–F). Subsequent colocalization analysis showed that WT TBCK exhibits modest colocalization with caveolin (MCC mean value: 0.32 ± 0.15) and GM130/GOLGA2 (MCC mean value: 0.37 ± 0.13) and high colocalization levels with STAM, RAB5A, COPII, and clathrin (MCC mean values: 0.76 ± 0.13, 0.74 ± 0.28, 0.79 ± 0.17, and 0.8 ± 0.13, respectively), consistent with an important role for TBCK in both endocytic and early secretory pathways (Figures 3A–F and Supplementary Figures 5A–F). Importantly, whereas a similar colocalization pattern was observed between TBCK-mutated protein and caveolin, COPII, and RAB5A (MCC mean values: 0.29 ± 0.23, 0.74 ± 0.26, and 0.67 ± 0.35, respectively) (Figures 3A,F and Supplementary Figures 5A–C), decreased colocalization was observed with clathrin (MCC mean value: 0.73 ± 0.14; *p* < 0.00001) (Figure 3B and Supplementary Figure 5D), and increased colocalization with STAM (MCC mean value: 0.83 ± 0.1; *p* < 0.0001) (Figure 3D and Supplementary Figure 5E) and GM130/GOLGA2 (MCC mean value: 0.49 ± 0.14; *p* < 0.0001) (Figure 3C and Supplementary Figure 5F).

We also examined the effect of lack of functional TBCK protein over clathrin, RAB5A, and STAM expression in iNPC grown as neurospheres (3D model system). RT-qPCR showed reduced clathrin heavy chain CLTD and STAM1 transcript levels in neurospheres of patients, compared with controls (Figure 3G). Interestingly, quantification of clathrin and STAM by Western blot showed a tendency to increase protein levels in patients but not statistically significant (Figure 3H and Supplementary Figures 4E,F). Together, these results suggest altered intracellular membrane trafficking along the endolysosomal and/or the early secretory pathways in patient-derived iNPC.

### Patient-Derived Neuroprogenitor Cells Do Not Show Evidence for Altered Endosome Maturation Along With the Endolysosomal System

As part of the ESCRT sorting machinery, STAM together with clathrin contributes to the recognition and sorting of ubiquitinated cargos at early endosomes, which mature to late endosomes prior to targeting ubiquitinated proteins for degradation at lysosomes (Vietri et al., 2020). Thus, to investigate whether altered colocalization of TBCK-mutated protein with STAM and clathrin is associated with defects in the endolysosomal system, we quantified the immunofluorescence intensities of RAB7A, a late-endosome marker. RAB7A fluorescence intensities did not differ between patients and controls (Supplementary Figure 6), suggesting that the expression of TBCK-mutated protein does not disrupt late endosome maturation in cells under normal growth conditions.

### Lack of Functional TBCK Protein May Alter Early Secretory Pathway and Autophagosome Membrane Recruitment in Basal Autophagy

Next, we sought to verify if the patient-derived cells show impairment of the early secretory pathway. STAM has been implicated in the deubiquitination of SEC31A (component of COPII outer layer), modulating the formation of large COPII carriers that mediate the transport of macromolecules (such as procollagen) from the endoplasmic reticulum (ER)-to-Golgi (Rismanchi et al., 2009; Kawaguchi et al., 2018). Thus, we first investigated whether patient-derived iNPC present abnormal large COPII-carrier formation (> 1 μm). Measurement of the diameter of COPII puncta showed that iNPC of patients had a smaller average diameter compared with controls (average diameter of large COPII carriers—patients: 1.89 ± 1.01 μm and controls: 2.31 ± 1.23 μm; *p* < 0.0001) (Figure 3I) and a tendency to have less large COPII carriers per cell (Figure 3J).

**FIGURE 4:**
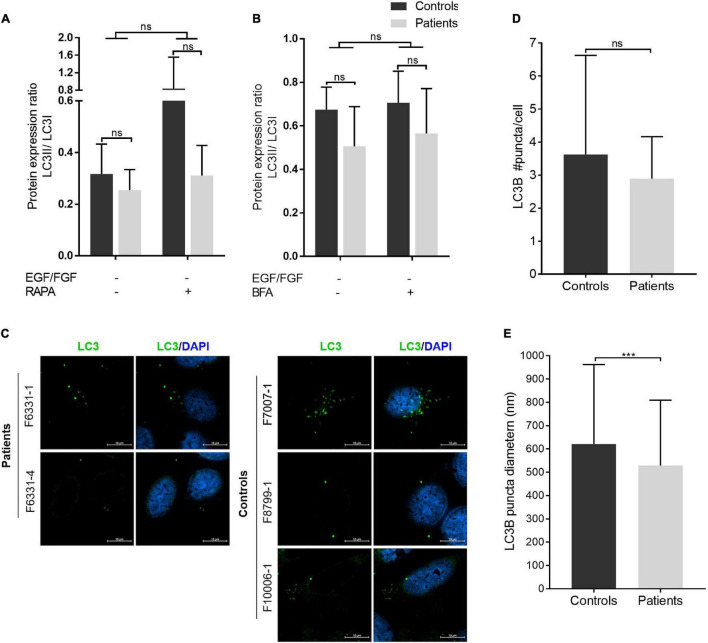
Autophagosome biogenesis analysis: LC3B puncta diameter is reduced in patient-derived iNPC. **(A,B)** Graphs show the densitometries of immunoblots shown in Supplementary Figure 4. Patients are represented in gray ray bars and controls in black bars. Graphs show the LC3A-B II/I ratio from iNPC cultivated under normal growth conditions and under stress-inducing conditions: absence of EGF and fibroblast growth factor-2 (FGF-2) for 24 h and **(A)** concomitant treatment with rapamycin (100 nM) for 24 h, and **(B)** subsequent treatment with BFA (4 μM) for 3 h. β-actin was used as a loading control. Graphs are represented in arbitrary units. **(C–E)** Immunostaining analysis of endogenous LC3B by confocal microscopy. Representative images are presented in panel **(C)**; average LC3B puncta diameter (nm) in panel **(D)**; and an average number of LC3B puncta per cell in panel **(E)**. ***p-value < 0.001; n.s.—not significant. Data are shown as mean values ± SD of biological replicates. Patients: F6331-1 and F6331-4 (*N* = 2); controls: F7007-1, F8799-1, and F10006-1 (*N* = 3).

COPII-coated vesicles have also been shown to act as templates for LC3 lipidation (conversion of cytoplasmic LC3I to membrane-bound LC3II) during autophagy, playing an important role as a membrane source for autophagosome biogenesis (Ge et al., 2014; Farhan et al., 2017; Shima et al., 2019). Thus, we also evaluated whether iNPC of patients show evidence of defective autophagosome biogenesis, by quantifying the protein levels of the autophagosome marker LC3 under normal culture condition (basal autophagy) and the three stress-inducing conditions (absence of EGF/FGF-2, and absence of EGF/FGF-2 + rapamycin or BFA). Western blot analysis of cells in all conditions showed a modest decrease in the LC3II/I ratio in iNPC of patients but not statistically significant (Figures 4A,B and Supplementary Figures 4G–J). Several other autophagy markers, including BECN1, p62/SQSTM1, and cathepsin D, did not show differences between patients and controls in the absence of EGF/FGF-2 only (Supplementary Figure 7). Despite the lack of statistical significance, the LC3II/I ratio always shows a trend to be decreased in iNPC of patients. Further confocal microscope analysis of LC3B under normal growth conditions showed that the number of LC3B puncta is also trended to be reduced in cells of patients, while the average diameter of LC3B puncta was significantly smaller compared with controls (LC3B average puncta diameter—patients: 528.7 ± 280.6 nm and controls: 621 ± 341.2 nm; *p* < 0.001) (Figures 4C–E). These results suggest altered autophagosome formation in basal autophagy in patient-derived iNPC.

### Neuroprogenitor Cells Expressing TBCK-Mutated Protein Show Altered Cell Cycle Progression and Severe Impairment in the Capacity of Cell Migration

**FIGURE 5:**
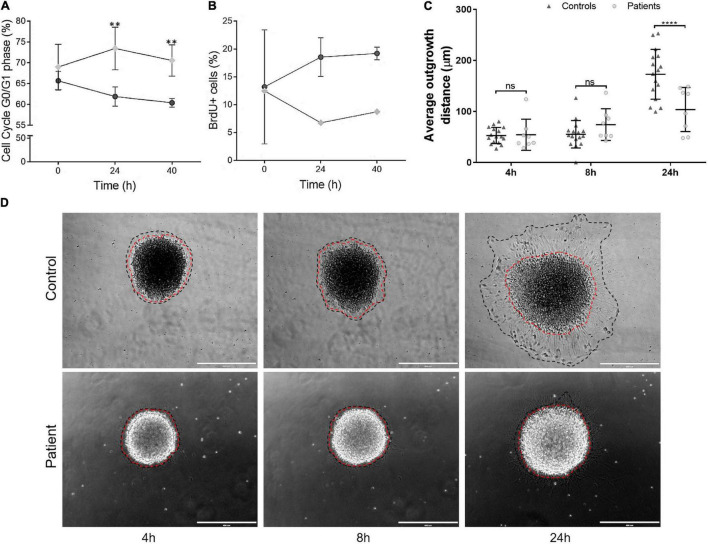
iNPC expressing TBCK-mutated protein show altered cell cycle progression, cell proliferation, and cell migration. **(A)** G0/G1-phase analyses at three time points, showing an increased percentage of cells of patients arrested at G0/G1 cell cycle stage. Cell cycle experiments were replicated twice. **(B)** BrdU-positive cells showed that iNPC of patients had lower incorporation of BrdU. Data are shown as percentages. BrdU incorporation experiment was performed once for each sample. Patient: F6331-1; controls: F7007-1, F8799-1, and F10006-1. **(C,D)** iNPC migration from neurospheres, measured as the distance of the longest outer diameter of the cells that migrated from the neurosphere, normalized to the inner neurosphere edge. **(D)** Representative images of migration assay. Red dashed line—inner diameter; black dashed line—outer diameter. Scale bar = 400 μm. Patients: F6331-1 and F6331-4 (*N* = 2); controls: F7007-1, F8799-1, and F10006-1 (*N* = 3); Neurosphere technical replicates = 4. Data are shown as mean values ± SD of patients and control biological replicates. **p-value < 0.01, ****p-value < 0.0001, ns - not significant.

Finally, we sought to verify whether the expression of TBCK-mutated protein is associated with aberrant cell proliferation and migration in the context of neural development. Flow cytometry analysis showed a higher percentage of cells arrested at G0/G1 in patient-derived iNPC at 24 h and 40 h of recovery from the withdrawal of growth factors compared with control iNPC (*p*-value < 0.01) (Figure 5A and Supplementary Figure 8). Detection of S-phase cells *via* BrdU incorporation showed that BrdU-positive cells in the control population increased on average 4.5 and 6.7% after 24 h and 40 h of medium supplementation, respectively, while BrdU-positive cells in the TBCK-mutated population decreased 12.6 and 6.8% after 24 h and 40 h of medium supplementation, respectively (Figure 5B). These alterations were not attributed to an increase in apoptosis since activation of caspase 3/7 did not differ between patient and control cells (Supplementary Table 4). Taken together, these results suggest that patient-derived iNPC show reduced cell proliferation due to delayed cell cycle progression.

We also evaluated the migration rates of iNPC from IHPRF3 patients and controls by quantifying the distance of cell migration outward from the edge of neurospheres at 4, 8, and 24 h after adhesion. While iNPC from control neurospheres showed robust migration rates after 24 h, we observed that patient-derived iNPC showed dramatically reduced capacity of migration (Figures 5C,D).

## Discussion

In this study, we described two sisters who were referred to the CEGH-CEL at the age of 3 and 6 years due to suspected ASD. Detailed clinical investigation and follow-up for 7 years revealed several facial dysmorphic features and progressive muscle weakness and psychomotor developmental delay. These clinical symptoms along with the biallelic LoF variants in *TBCK* led to the diagnosis of IHPRF3 (OMIM#616900), an autosomal recessive condition with approximately 40 cases reported so far. Several cases of IHPRF3 show premature death before early adulthood. Importantly, in keeping with another patient described by Bhoj et al. (2016), we highlighted autism as an additional clinical feature of the syndrome.

Our patients are compound heterozygous for pathogenic LoF variants in *TBCK*: a stopgain variant (p.Tyr710*) in exon 23 and a microdeletion (p.Glu687Valfs9*) predicted to create a premature stop codon in exon 24 that has been previously identified in homozygosity in another IHPRF3 patient (Sumathipala et al., 2019). While most pathogenic variants reported in IHPRF3 patients located at the region encompassing the kinase and TBC domains (Zapata-Aldana et al., 2019), the variants in this study identified, together with two variants reported elsewhere (Bhoj et al., 2016; Sumathipala et al., 2019), located in a more distal region, downstream of the TBC domain.

The hypomorphic variants in *TBCK* in this study reported led to reduced mRNA and protein expression levels in patient-derived iNPC; however, we found a high number of TBCK-positive puncta in iNPC of patients through confocal microscope immunofluorescence labeling, with the same subcellular localization as iNPC of controls. Previous studies on fibroblasts and lymphoblastoid cells of IHPRF3 patients, harboring variant upstream of the TBC domain, also showed a drastic reduction to an absence of TBCK protein in cells of patients (Bhoj et al., 2016; Sumathipala et al., 2019). These results may suggest that TBCK in patient-derived iNPC partly escapes from both nonsense mediated-mRNA decay and protein quality control pathways (Giannandrea et al., 2013; Supek et al., 2021). Similar findings were described for odontochondrodysplasia (OCDC) (Wehrle et al., 2019). OCDC patients, who harbored hypomorphic mutations in the thyroid hormone receptor interactor 11 (TRIP11), also known as Golgi-associated microtubule-binding protein (GMAP-210), expressed different mutant isoforms of this gene, which can maintain the partial function of the wild-type isoforms. Thus, we can speculate that the clinical progression of the syndrome in our patients, which are milder than most of the patients reported so far (Beck-Wödl et al., 2018; Sumathipala et al., 2019; Zapata-Aldana et al., 2019), may possibly be due to a higher expression of mutant *TBCK*.

Neuroprogenitor cells from our patients showed reduced RPS6 phosphorylation when submitted to the withdrawal of EGF and FGF-2 growth factors either with or without rapamycin treatment, indicating reduced mTOR signaling pathway activation. Conversely, the addition of BFA, which interferes with glutamine-induced lysosomal localization and activation of mTORC1 (Jewell et al., 2015; Meng et al., 2020), to growth factor-deprived cells led to similar RPS6 phosphorylation levels between patients and controls. That is, BFA seems to have affected iNPC of controls in a more pronounced way than iNPC of patients. Previous study also demonstrated reduced mTOR signaling pathway on TBCK depleted non-neuronal cells (Liu et al., 2013; Bhoj et al., 2016; Ortiz-González et al., 2018), which could be partially rescued by leucine treatment. mTOR signaling activation by leucine depends on Rag GTPase (Lee et al., 2018), which does not seem to be dysregulated in IHPRF3 patients. In contrast, our results may suggest that TBCK plays a role in mTOR signaling through the same pathway as BFA, and preexisting disruption of this signaling in iNPC of patients makes cells of patients less sensitive to the inhibitory effect of BFA compared with controls. BFA targets the GEF of intra-Golgi vesicle transport regulator ARF1 GTPase, preventing the conversion of ARF1 from its GDP-bound state to the active GTP-bound state (Robineau et al., 2000). ARF1 activity toward mTOR signaling pathway is induced by a specific subset of amino acids, including asparagine and glutamine, with the latter being highly enriched in the NB medium (Jewell et al., 2015; Meng et al., 2020). Thus, reduced RPS6 activation in iNPC expressing TBCK-mutated protein may be due to impaired ARF1 activation making patient-derived iNPC less sensitive to an inhibitory effect of BFA.

Consistent with a role in endosomal and/or early secretory vesicle transport regulation, we showed that TBCK protein in iNPC under normal growth conditions localizes to both the endosomes (e.g., RAB5A^+^ compartment) and the early secretory pathway (e.g., COPII vesicles). In fact, knockdown of TBCK has been suggested to affect vesicle transport at RAB5^+^/EEA1^+^ early endosomes in non-neural cells (Collinet et al., 2010). However, in this study, iNPC of patients did not show altered endosome maturation.

Notably, our results provide support for the interaction between TBCK and STAM, which have been predicted to act as a complex, together with HGS and RANGRF in HeLa and HEK293 cell lines (Havugimana et al., 2012). Increased colocalization of TBCK-mutated protein with STAM and GM130/GOLGA2 (a *cis*-Golgi tethering factor that facilitates ER-derived vesicle fusion) and decreased colocalization with clathrin observed in cells of patients suggest altered early secretory transport and impaired clathrin-coated vesicle-dependent post-Golgi transport of proteins. In agreement with this view, patient-derived iNPC showed a smaller average diameter of COPII carriers, suggesting impaired macromolecule transport from ER to Golgi. Consistent with early secretory defects, iNPC of our patients showed smaller LC3B puncta. Despite the lack of statistical significance, which may be due to the limited detection capability of Western blot, the average LC3II/I ratio tends to be decreased in iNPC of patients, in both basal and stress-induced autophagy. Thus, further study is needed to elucidate the autophagy regulation in iNPC with depletion of functional TBCK. Regardless, our findings support the hypothesis that TBCK acts in ER-to-Golgi vesicle trafficking and that lack of functional TBCK protein might impair both the transport of newly synthesized proteins to their destination and autophagosome biogenesis due to altered early secretory transport (Supplementary Figure 9). It is important to note that unlike observations by Ortiz-González et al. (2018) reduced mTOR signaling in iNPC of patients treated with the well-established autophagy inducer, rapamycin, was not associated with an increased level of autophagosome formation. This apparent contradictory data may be associated with previous suggestions that TBCK seems to present functional differences according to the cell type (Liu et al., 2013; Wu et al., 2014; Wu and Lu, 2021) and emphasize the relevance of studying neural cells to investigate the pathophysiological mechanisms of neuronal phenotypes of IHPRF3. Furthermore, the discordance between mTOR signaling activity and autophagosome formation observed in cells of our patients suggests that dysregulated mTOR signaling alone may be insufficient to account for all the clinical features of the syndrome.

Furthermore, in this study, we observed that patient-derived iNPC showed G0/G1 cell cycle arrest and delayed S-phase progression, as well as a significantly impaired capacity of migration. Impaired cell cycle progression has also been observed by Wu et al. (2014). These authors together with Liu et al. (2013) observed an overlapped localization between TBCK and α- and γ-tubulin, respectively, suggesting a potential role of TBCK on microtubule (MT) nucleation. In this context, studies have shown that Golgi apparatus can function as an important microtubule-organizing center (MTOC) in many cell types, and unlike centrosomal MTOC, it can give rise to polarized MTs important for a number of cellular processes, including Golgi reassembly after mitosis (Maia et al., 2013) and polarized transport of post-Golgi carriers that are important for cell migration (Vinogradova et al., 2009, 2012; Hurtado et al., 2011; Wu et al., 2016). In neuronal cell types, Golgi-anchored MTs have also been implicated in neurite outgrowth and branching (Oddoux et al., 2013; Yalgin et al., 2015). Thus, it is tempting to speculate that impaired activity of TBCK mutant protein over other Golgi resident vesicle transport regulators impact MT nucleation and Golgi-derived MT dependent processes that are essential for normal brain development and structural organization, such as cell division, migration, and neuronal morphogenesis (Ori-McKenney et al., 2012; Etienne-Manneville, 2013; Roccio et al., 2013; Borrell and Calegari, 2014; Maizels and Gerlitz, 2015; Garcin and Straube, 2019; Shokrollahi and Mekhail, 2021). However, we cannot rule out that the migration defect observed in the TBCK-deficient neurospheres may also be related to the altered proliferation of cells of patients. Nevertheless, impairment in these processes may further contribute to abnormal brain structures, like microcephaly and cortical atrophy presented by IHPRF3 patients.

In summary, we described a novel IHPRF3 family, adding autism as a clinical feature of this syndrome. Using iNPC from IHPRF3 patients and control individuals, we pinpointed a role for *TBCK* in regulating the early secretory pathway and suggested that the impairment in mTOR signaling and autophagosome biogenesis observed in *TBCK*-mutated cells might be related to impaired signaling toward other early secretory transport regulators, such as RAB1A or ARF1 small GTPases. It is of note that pathogenic mutations in other members of the TBC family of proteins (*TBC1D20*, *TBC1D23*, *TBC1D24*), which also mediate intracellular membrane transport, such as endosome-to-Golgi and ER-to-Golgi trafficking or autophagy, have also been implicated in neurodevelopmental syndromes (OMIM#615663, OMIM#617695, and OMIM#220500, respectively) with high clinical overlap with IHPRF3 (Falace et al., 2014; Sidjanin et al., 2016; Ivanova et al., 2017; Shin et al., 2017; Aprile et al., 2019), reinforcing the importance of vesicle trafficking kinetics in neurodevelopment.

## Data Availability Statement

The datasets presented in this study can be found in online repositories. The names of the repository and accession numbers can be found below: https://www.ncbi.nlm.nih.gov/ (clinvarSCV002032078 and SCV002032079).

## Ethics Statement

The studies involving human participants were reviewed and approved by National Ethics Committee (Comissão Nacional de Ética em Pesquisa no Brazil, Process no. CAAE43559314.0.0000.5464). Written informed consent to participate in this study was provided by the participants’ legal guardian/next of kin. Written informed consent was obtained from the individual(s), and minor(s)’ legal guardian/next of kin, for the publication of any potentially identifiable images or data included in this article.

## Author Contributions

DPM, AMS, and MP-B: conceptualization, methodology, and writing—original draft. ATS, EV-B, MF, MRF, RC, MM, NL, KG-O, EZ, DB, KW, ML, HN, GSK, and ALS: investigation. DPM, AMS, GSK, ALS, and MP-B: writing—review and editing. MP-B: funding acquisition, resources, and supervision. All authors contributed to the article and approved the submitted version.

## Conflict of Interest

The authors declare that the research was conducted in the absence of any commercial or financial relationships that could be construed as a potential conflict of interest.

## Publisher’s Note

All claims expressed in this article are solely those of the authors and do not necessarily represent those of their affiliated organizations, or those of the publisher, the editors and the reviewers. Any product that may be evaluated in this article, or claim that may be made by its manufacturer, is not guaranteed or endorsed by the publisher.

## Footnotes

1
https://genome.ucsc.edu/

2
http://www.ncbi.nlm.nih.gov/tools/primer-blast/

3
http://rsbweb.nih.gov/ij/
