# Supplementary material for: Neuroprogenitor Cells From Patients With TBCK Encephalopathy Suggest Deregulation of Early Secretory Vesicle Transport
Source: Front Cell Neurosci. 2022 Jan 13;15:803302. doi: 10.3389/fncel.2021.803302 (PMC8793280; doi:10.3389/fncel.2021.803302)
Supplement: Supplementary file 1 [file Data_Sheet_1.pdf]

Moreira DP, Suzuki M, et al.

Title: Neuroprogenitor cells from patients with TBCK encephalopathy suggest deregulation of early secretory vesicle transport

Supplementary Information

Supplementary Table 1: Primer pairs for TBCK sequencing.

Primer-pair-name	sense	antisense
TBCK-seq-gDNA	GCAAAACTTGCTACCTGGAGGA	TTCACTTAGGAGAGATGGTGTTT
TBCK-seq-cDNA	ATCTCTATGCCATCCCTTGGTT	ACACAAGTCAATCAGGTCCTCT

Supplementary Table 2: Primer pairs for analyses of intragenic CNV between exons 22 and 24 of TBCK. We used GAPDH as control.

Primer Pair	sense	antisense
TBCK_P1	TGTTGAAACACCTACATGGCATAG	ACTCCTCCAGGTAGCAAGTTTTG
TBCK_P2	CCCAAAATACATTCCGTTCAAAAAC	AGTGCCAAAGTCTTTCCCATCAG
TBCK_P3	GACCAAGGAGTGAGGGTCAGGG	GCTTCCGGTTATTAGCTCCAGCCC
TBCK_P4	GCAGTCCTTCAATCACTGGCATTGG	TGTGCTTTGGAAGGGTTTTGAGATG
TBCK_P5	GACACCTTCAAGTTGACAGCAC	GAAAGAGAGCAGCTGTCCAGGG
TBCK_P6	GCAGGAATCCTTTCACAAACCAG	TGTAACGTGATTCTAGGAAGCTG
GAPDH	TGCTCACATATTCTGGAGGAGC	TGTAAACCTGGGGGAATACGTG

Supplementary Table 3: Primer pairs for relative mRNA quantification (RT-PCR).

Target	sense	antisense
TBCK-mRNA-1	CTGCTCTTCTGGGAGTTGAGGGAG	CAGGATGAGACACTACCCAGGCTT
TBCK-mRNA-2	GTCTTTGTGCTTGCCTGCGTCC	GCTCCCATTTCAGCGTCCTTCAG
TBCK-mRNA-3	GGTCATTGTCATCGTGGGGCAT	GGCAGTCACACTCTTGGTTCTTCA
TBCK-mRNA-4	TCCTGTAGCAAAGACGAATGGC	TGTAAAGCAATGAGAGAAGGTAATGAGT
CLTC_mRNA	GCCATGCCCTATTTCATCCAG	TCAACTGGGGCTGACCATAAAC
CLTD-like_mRNA	CAGCGGGAAACCAACCTTT	ACCATGTTTAGCTCCAATCTGC
RAB5A _mRNA	AGACCCAACGGGCCAAATAC	TTGGGTTAGAAAAGCAGCCCC
STAM1 _mRNA	CAGTCTCGCACTGGACCTAA	CCTGCATAGCAACGTGAGGA
STAM2 _mRNA	GAGATGCCTTTGTTCACCGC	AGGCAATCTTTCGCTCCATT

Supplementary Table 4: Caspase 3 staining is not different between TBCK patients and healthy controls iNPC.

Individuals	Cells (mean±SD)	Casp3 (mean±SD)	p-value*
F6331-1	452,5±45,96	4,5±0,71	
F7007-1	362±42,43	1±1,41	0.178178823
F8799-1	329±49,5	1±1,41	0.12332093
F10006-1	334±1,41	3,5±3,54	0.17011571

* Wilcoxon-Mann-Whitney

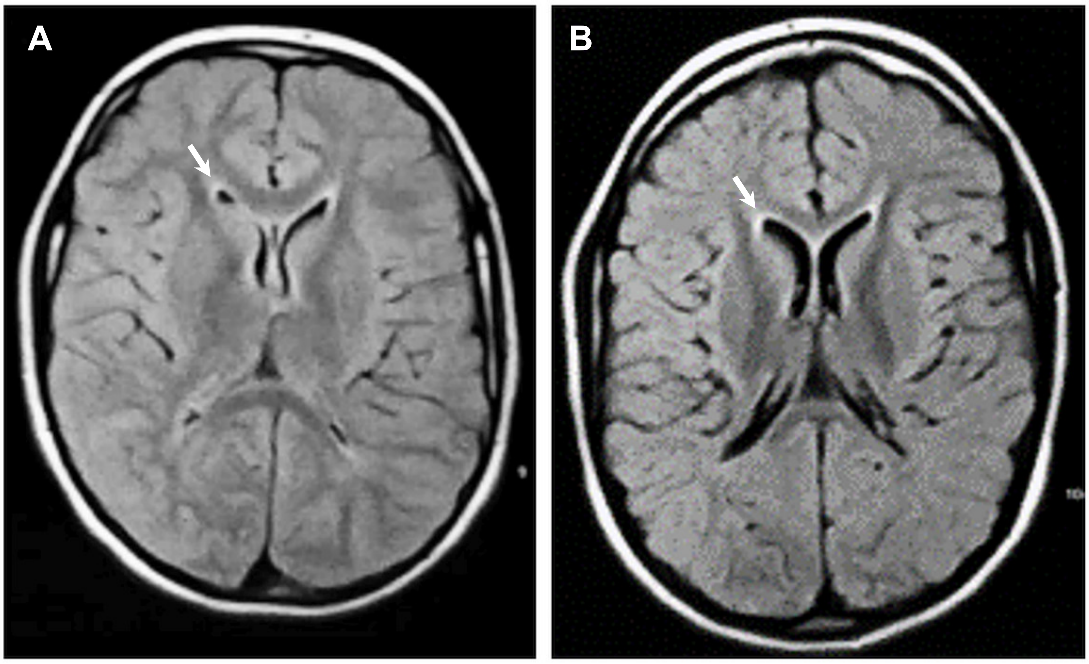

Supplementary Figure 1: Image of MRI axial plane in FLAIR sequence, showing that individuals 6331-1 (A) and 6331-4 (B) (MRI at 12 years and 9 years, respectively) have volumetric global reduction of brain and abnormal white matter hyperintesity (white arrow).

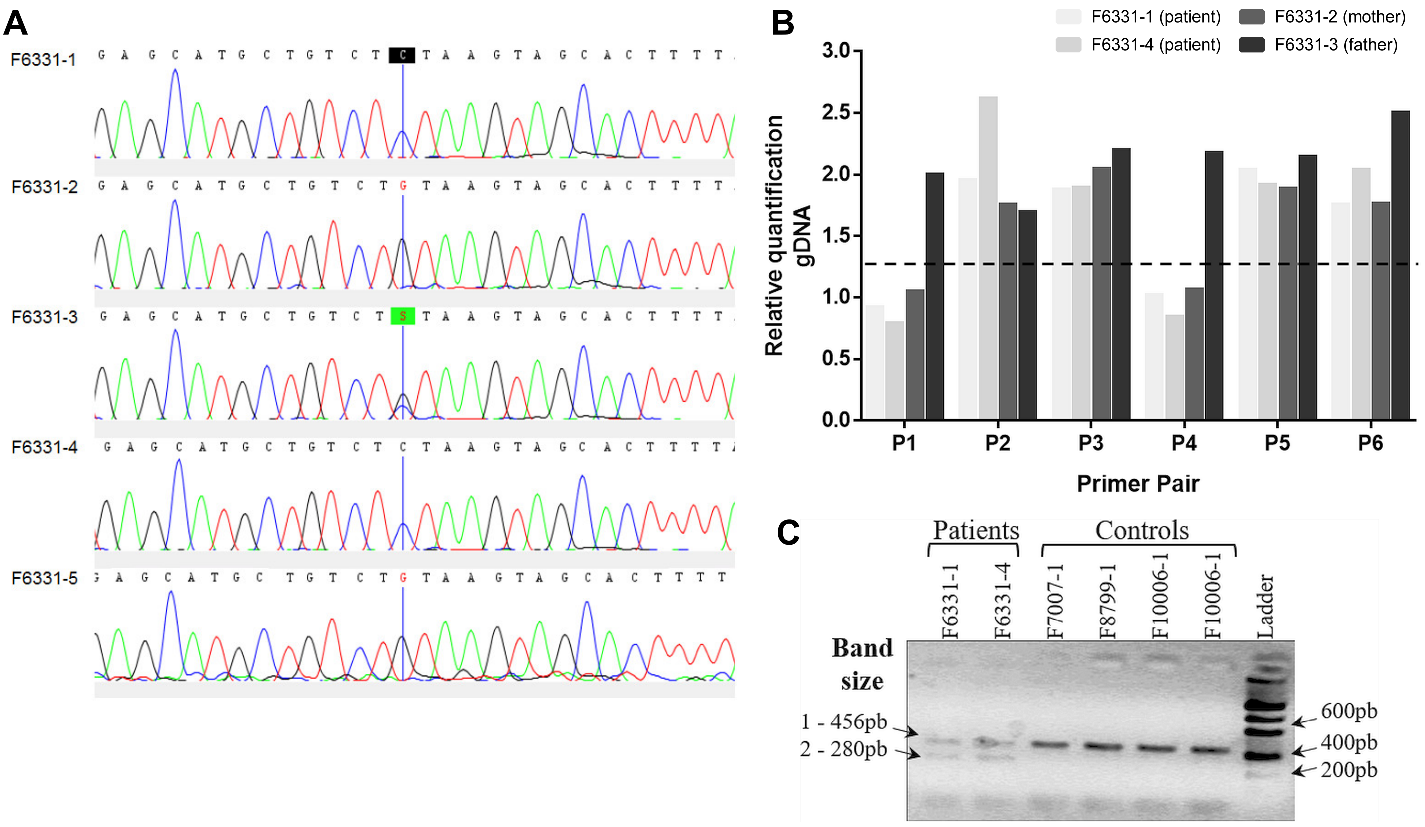

Supplementary Figure 2: Genomic analysis of *TBCK* variants.

A) Chromatograms of Sanger sequencing show the genomic stop codon variant in the members of the F6331 family. Both affected children (F6331-1 and F6331-4) harbor a mutation in one allele inherited from their father (F6331-3), who is heterozygous for the variant. F6331-2 (mother) and F6331-5 (healthy sister) show only wild-type allele. Blue vertical line indicates the variant position.

B) Relative quantification of genomic DNA (gDNA) exhibiting a deletion, involving primer pairs P1 and P4, shared among the affected children (F6331-1 and F6331-4) and their mother (F6331-2). A cutoff of < 1.3-fold (dashed line) was used to determine the allele with the microdeletion. The genomic position amplified by each primer pair is described in supplementary table 2.

C) Agarose gel showing two bands of cDNA's PCR. Band 1: 456pb – expected amplicon size of a normal allele; Band 2: 280pb – altered amplicon size from deleted allele). Low mass ladder was used as reference.

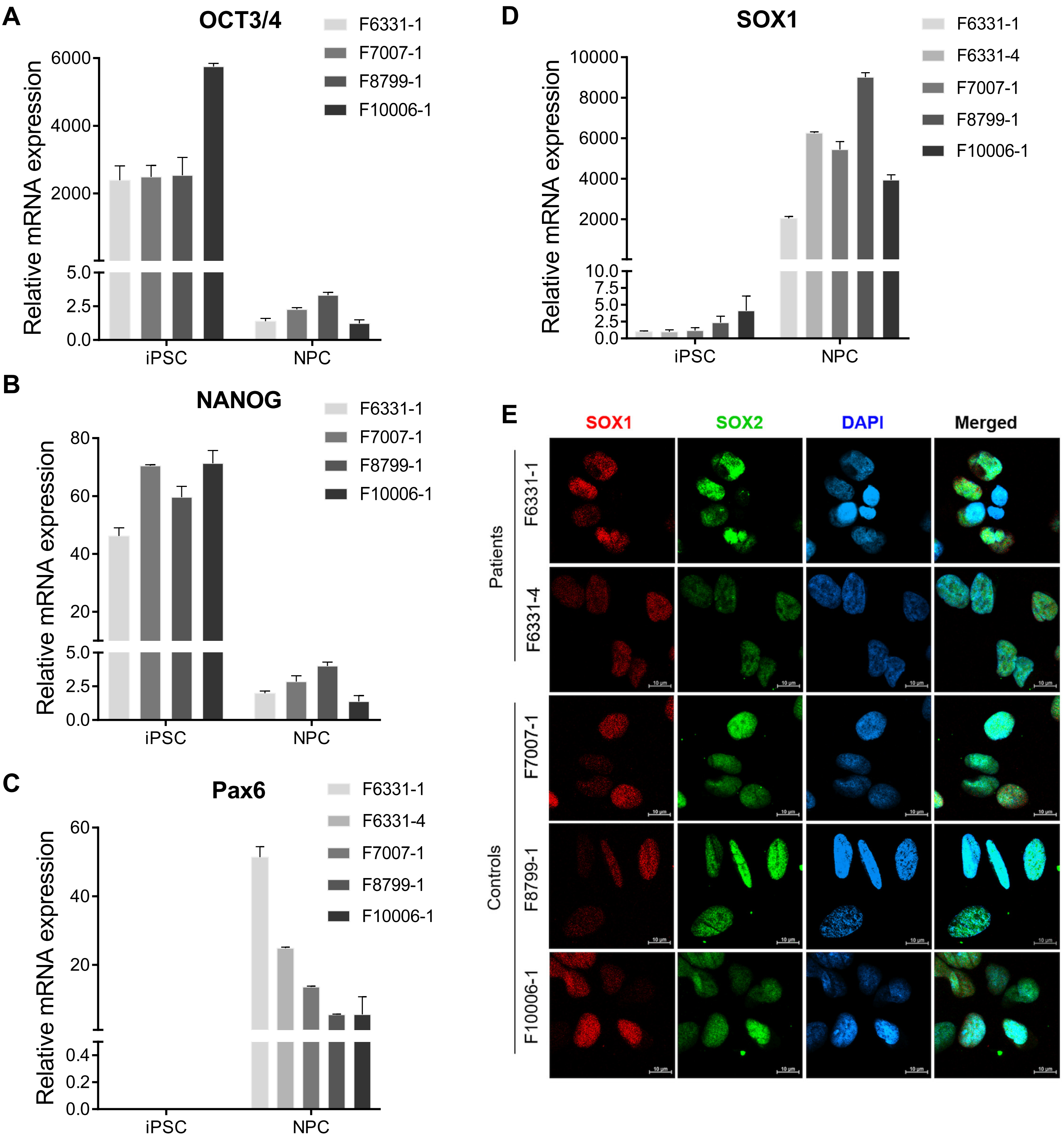

Supplementary Figure 3: iPSC and iNPC characterization.

A-D) mRNA expression levels of pluripotency markers, OCT3/4 and NANOG (A-B), and neural markers, PAX6 and SOX1 (C-D);

E) Immunostainning of two neural cell markers (SOX1 and SOX2).

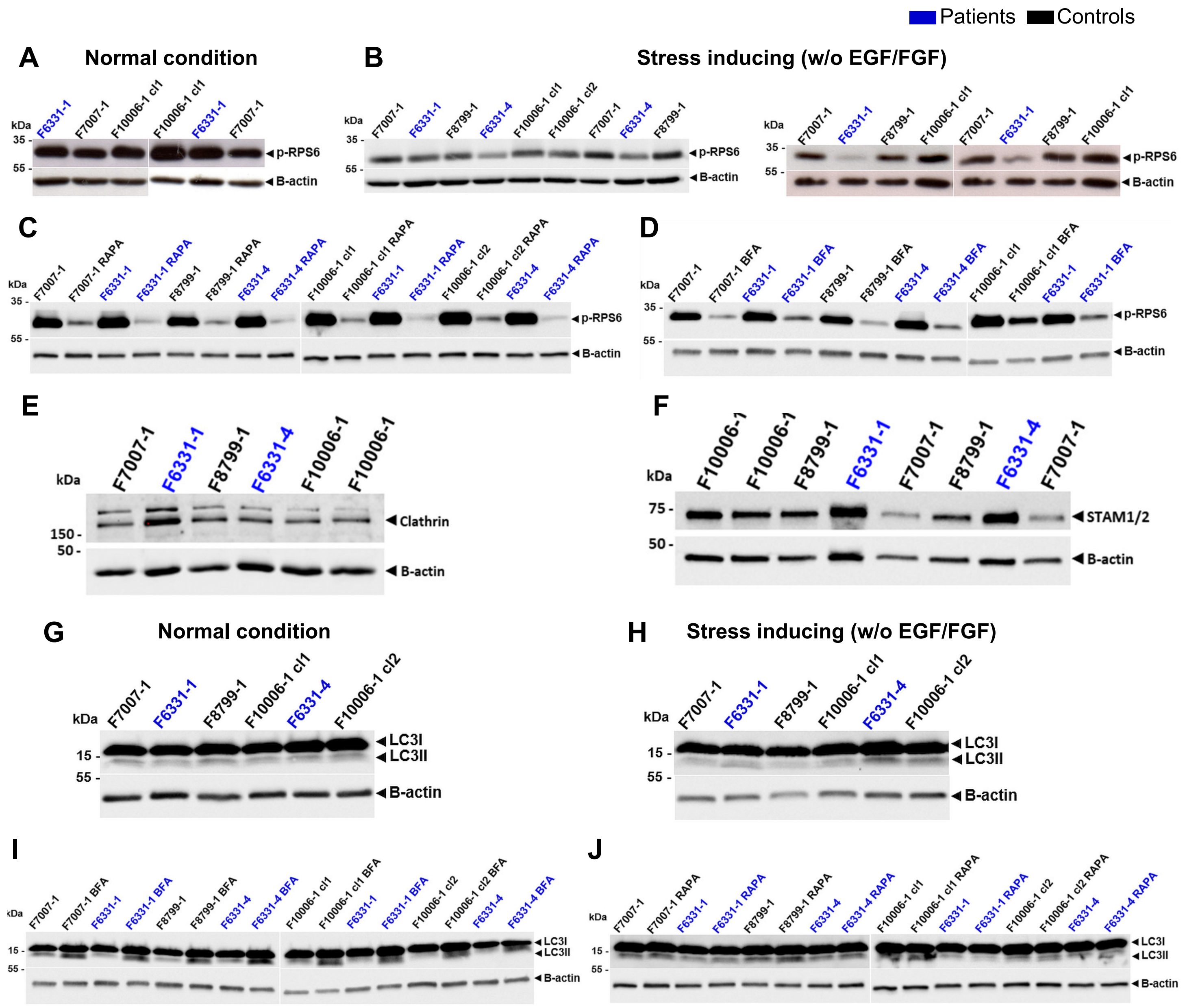

Supplementary Figure 4: Western blot for p-RPS6 and autophagosome marker LC3, in iNPC.

A-D) Blots of phospho-RPS6 of iNPC cultured in normal and stress inducing conditions;

E) Clathrin heavy chain and F) STAM1/2 of iNPC cultured under normal growth conditions

G-J) Blots of LC3A-BII/I of iNPC cultured in normal and stress inducing conditions.

Stress conditions were induced through: removal of EGF and FGF-2 for 24 hours only; removal of EGF and FGF-2 for 24 hours and addition of either rapamycin (RAPA – 100 nM) for 24 hours or brefeldin A (BFA – 4 μM) for 3 hours.

β-actin was used as a loading control. The molecular mass is indicated in kDa.

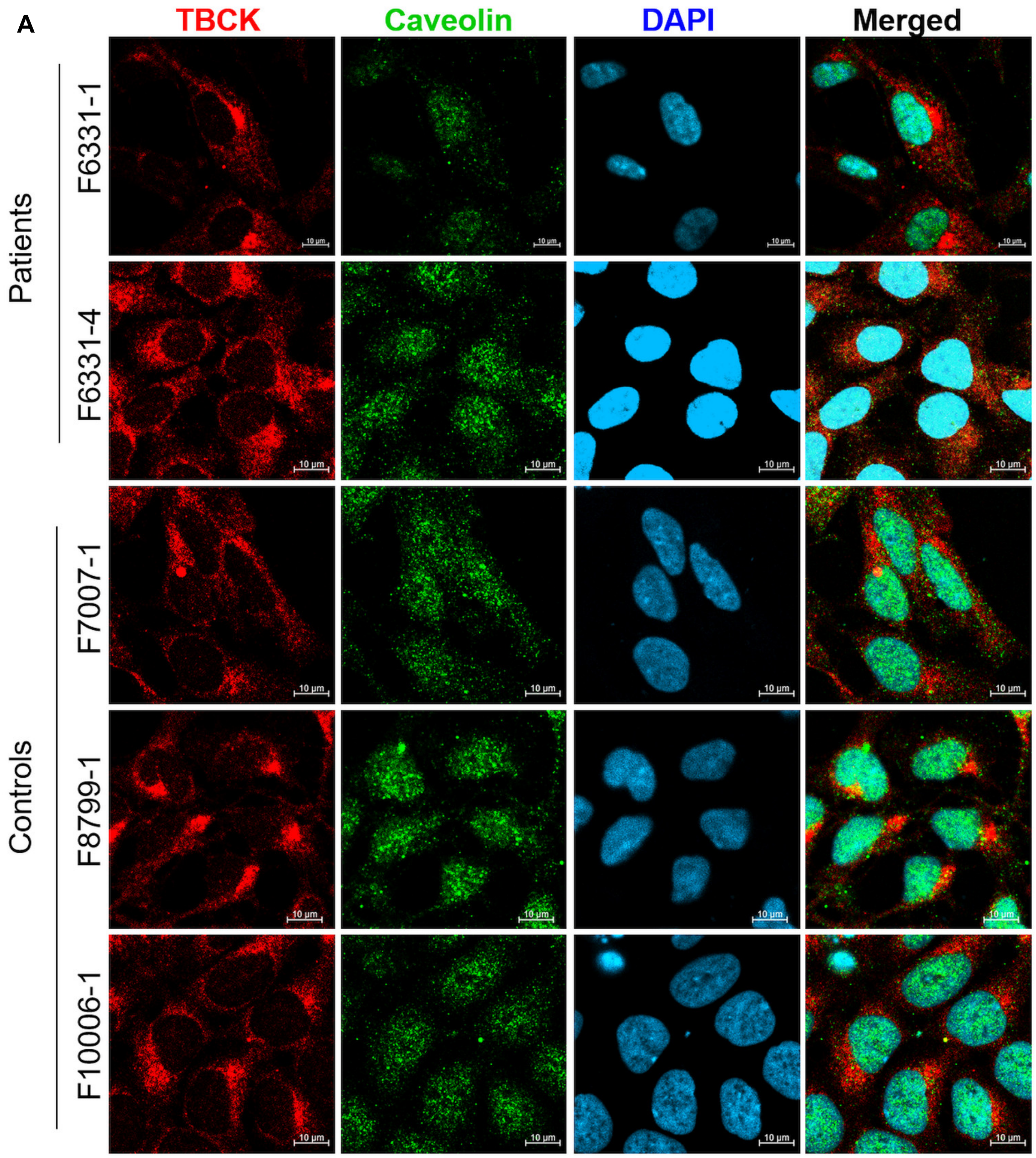

Supplementary Figure 5A: continue…

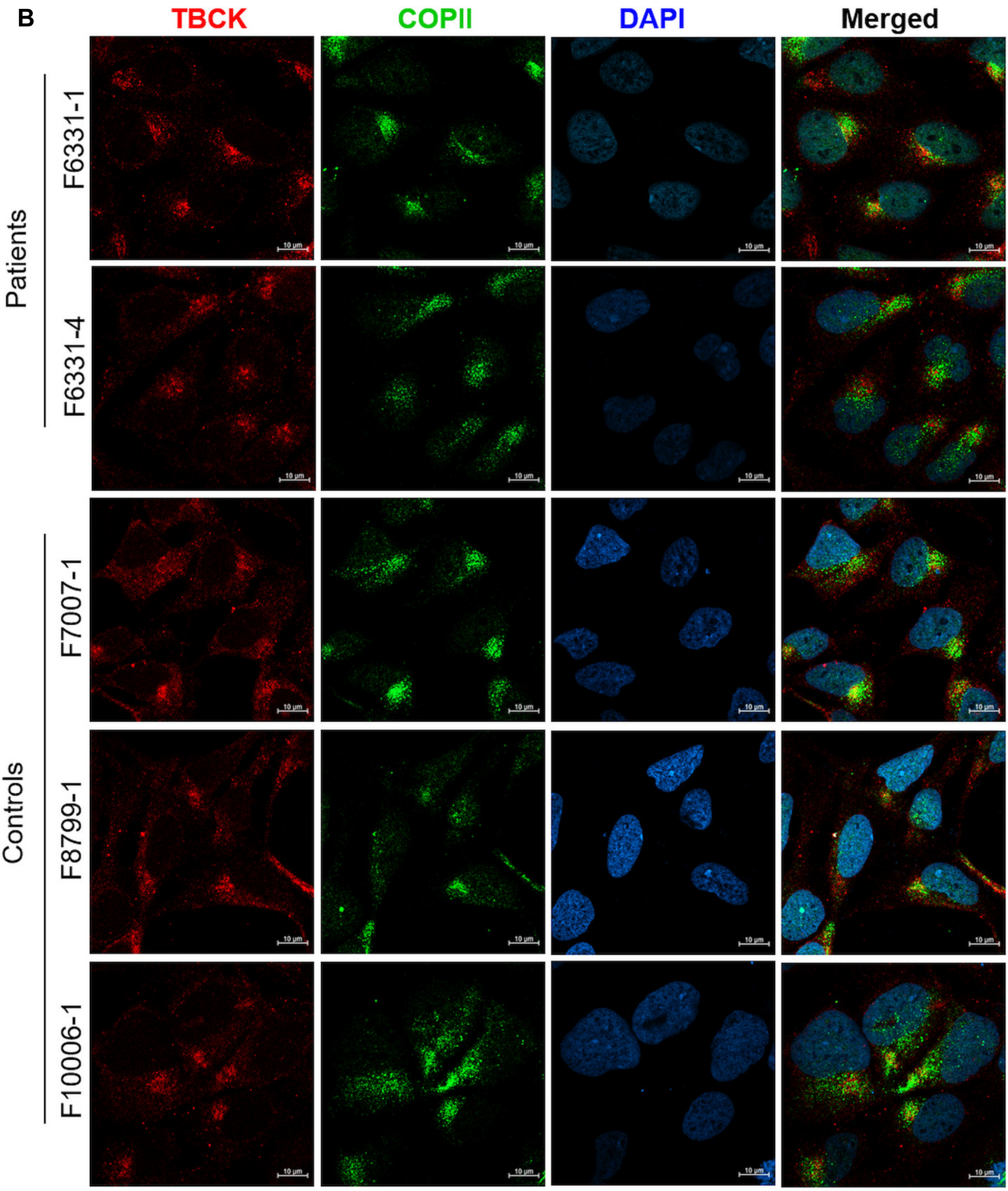

Supplementary Figure 5B: continue…

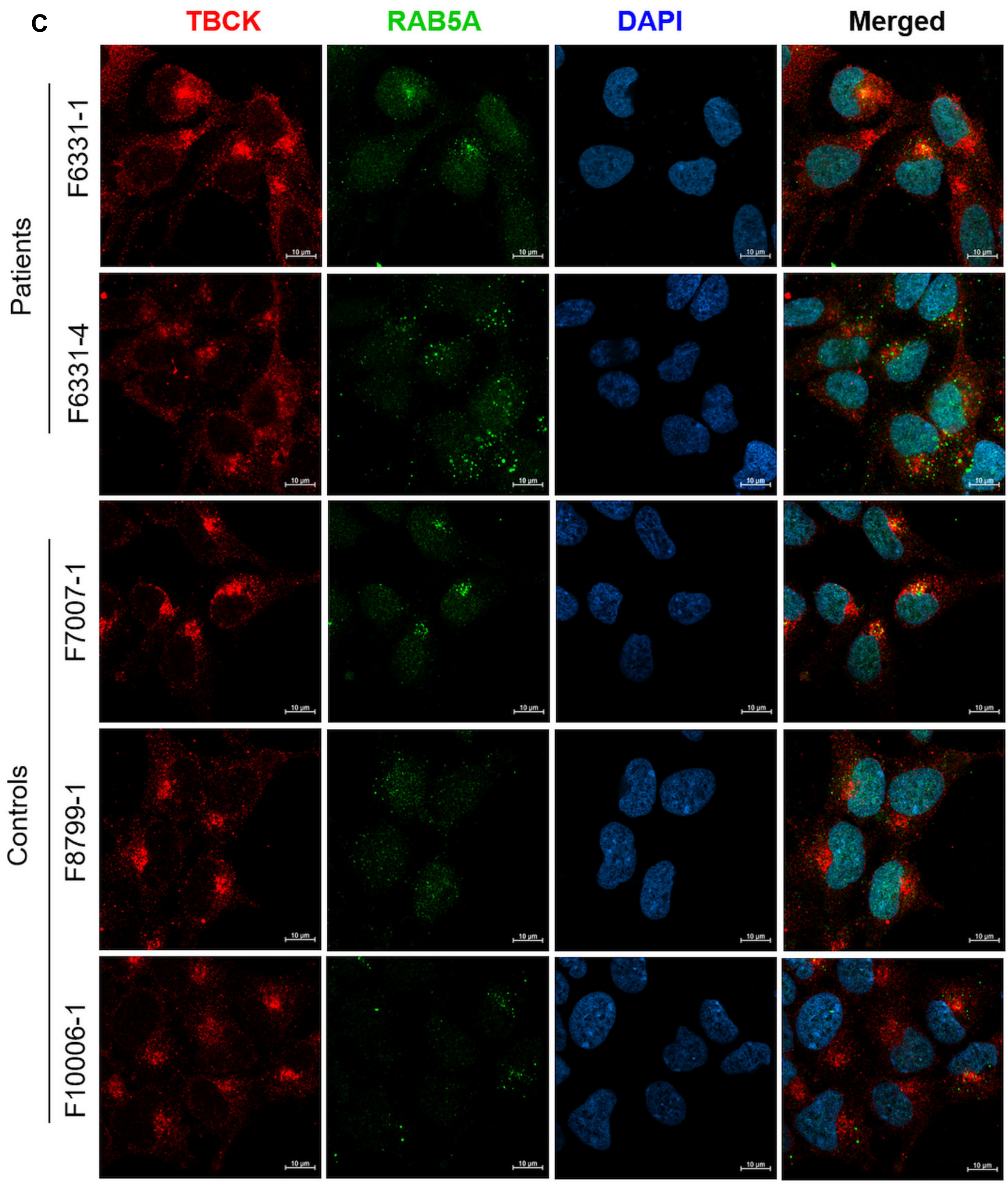

Supplementary Figure 5C: continue…

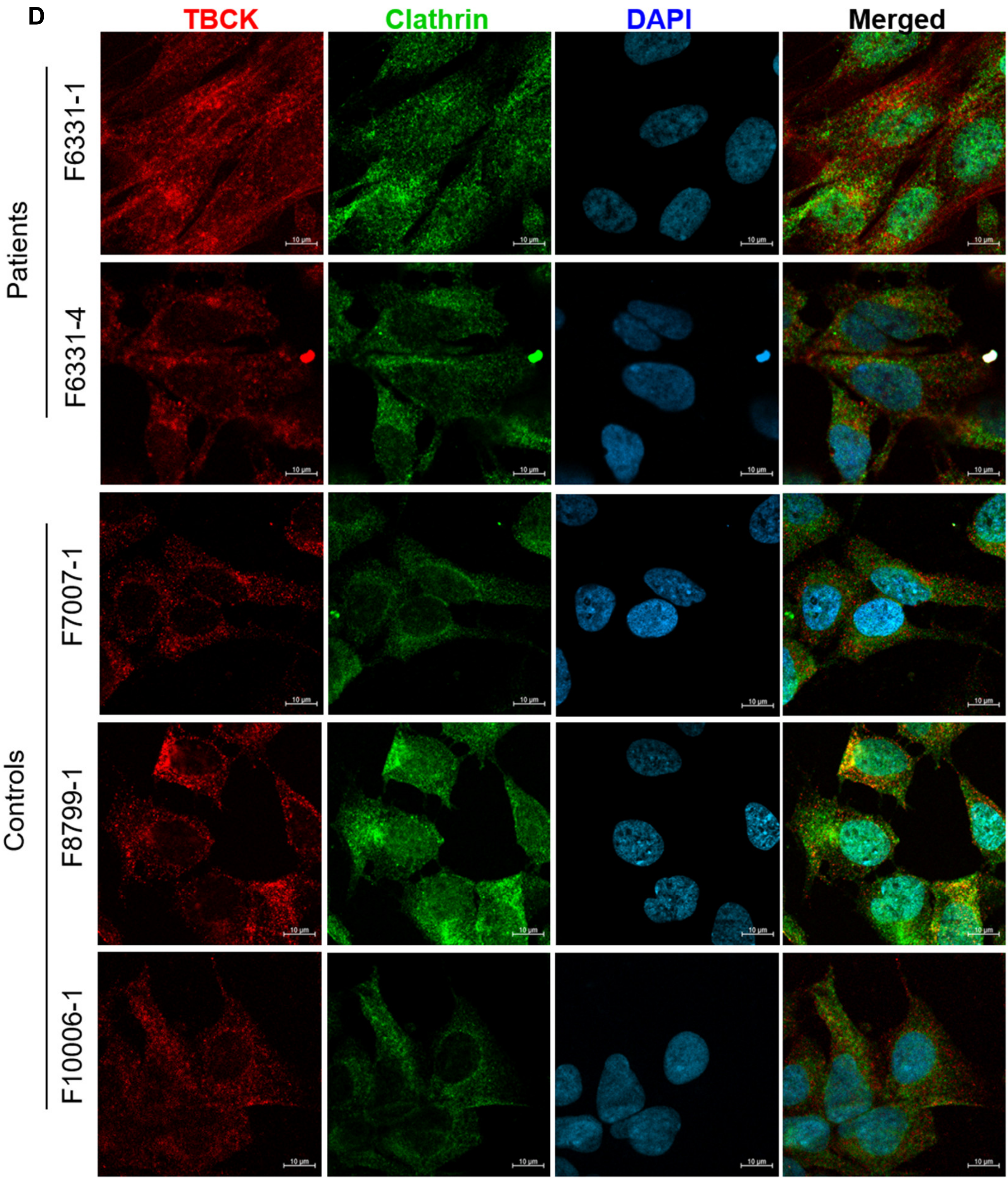

Supplementary Figure 5D: continue…

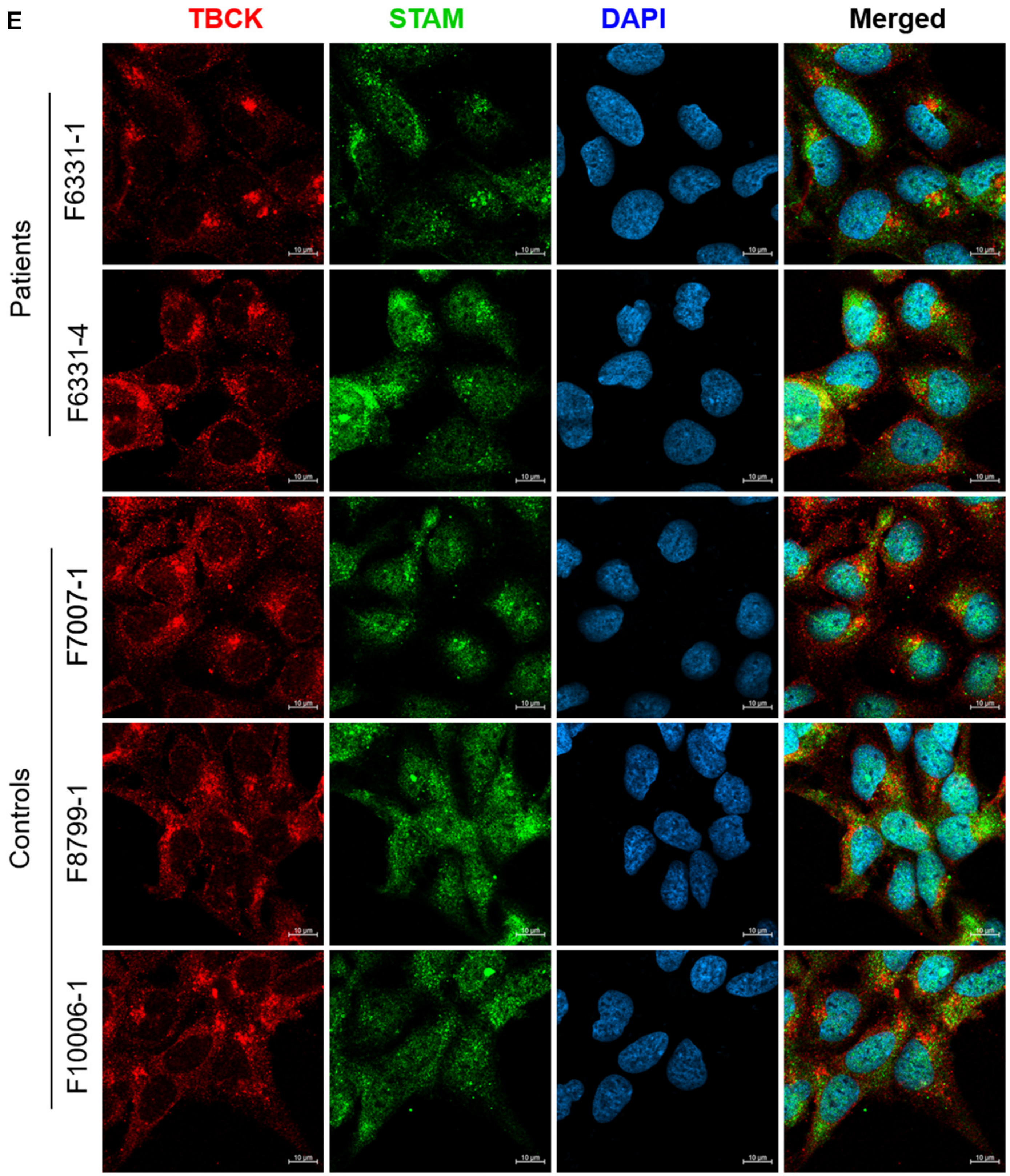

Supplementary Figure 5E: continue…

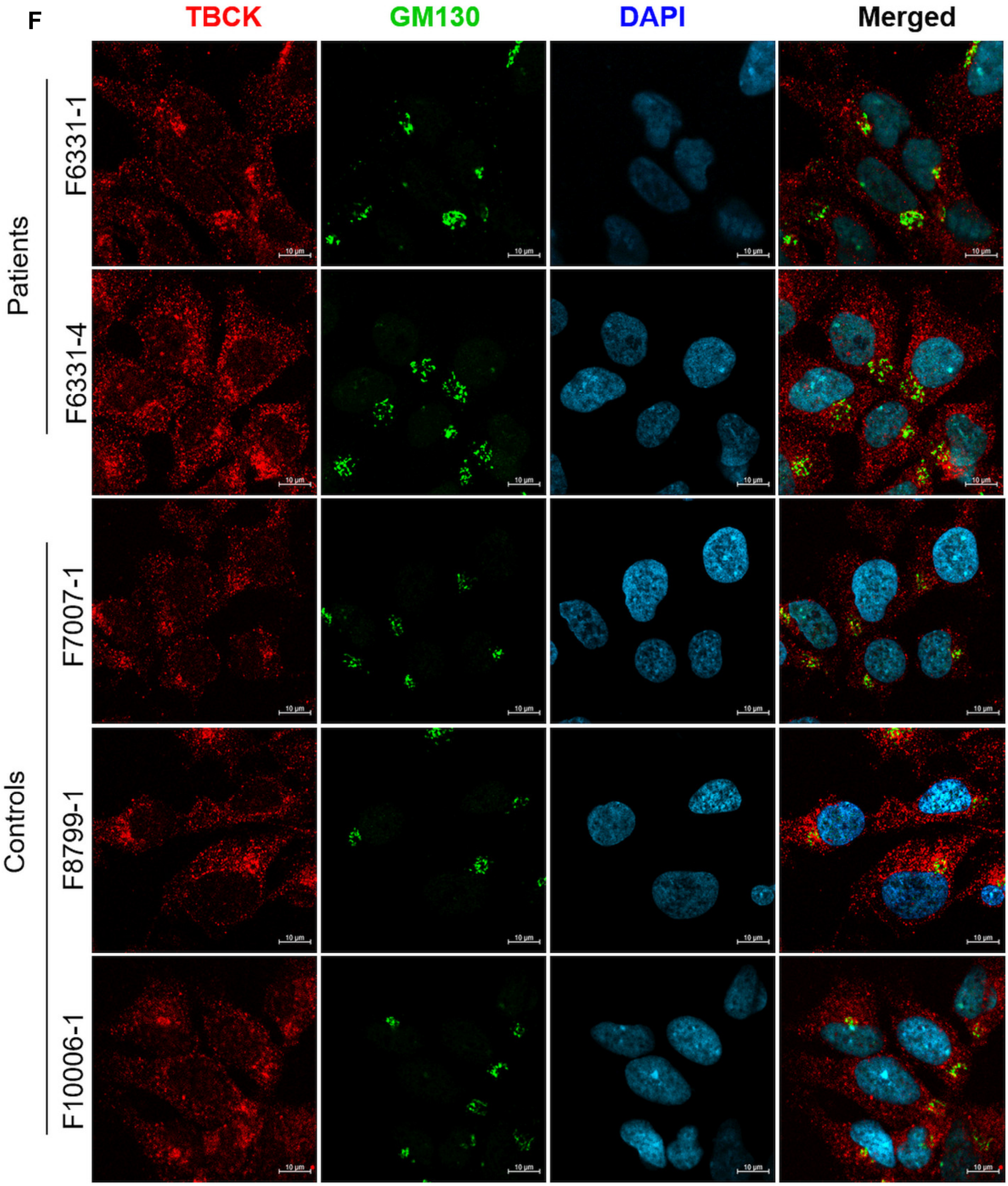

Supplementary Figure 5: Immunostainning of intracellular vesicle trafficking components and TBCK in iNPC. Colocalization of *TBCK* with: A) Caveolin; B) COPII, C) RAB5A, D) Clathrin; E) STAM; and F) GM130/GOLGA2. DAPI (blue) marks cell nuclei. Scale bar = 10 µM.

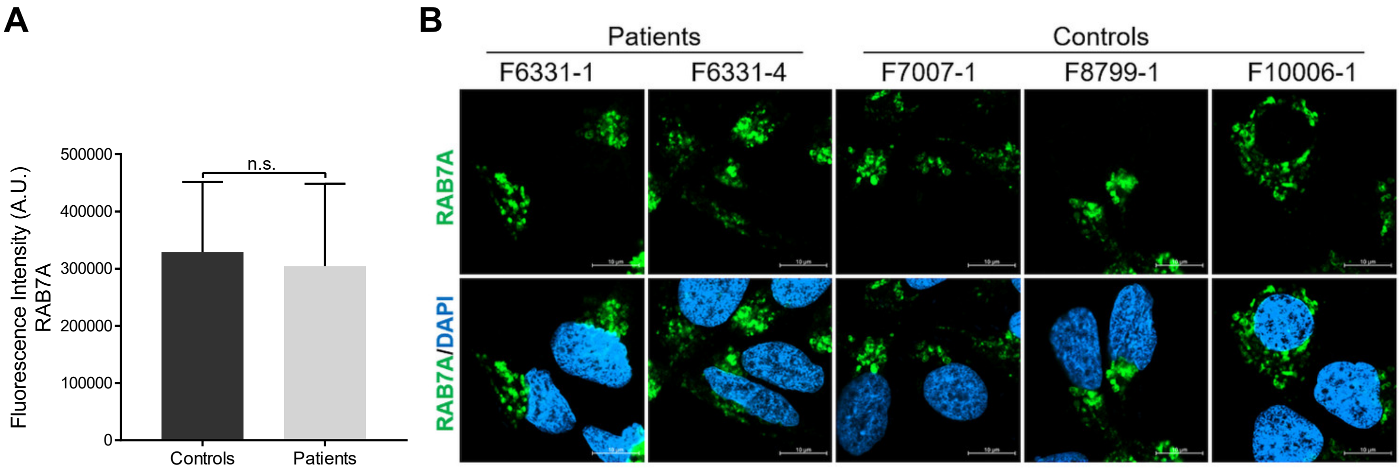

Supplementary Figure 6: Fluorescence immunostaining of RAB7A.

A) Graph showing quantification of RAB7A immunofluorescence intensities (arbitrary unit, AU);

B) Representative images showing RAB7A in iNPC. DAPI (blue) marks cell nuclei. Scale bar = 10 μM.

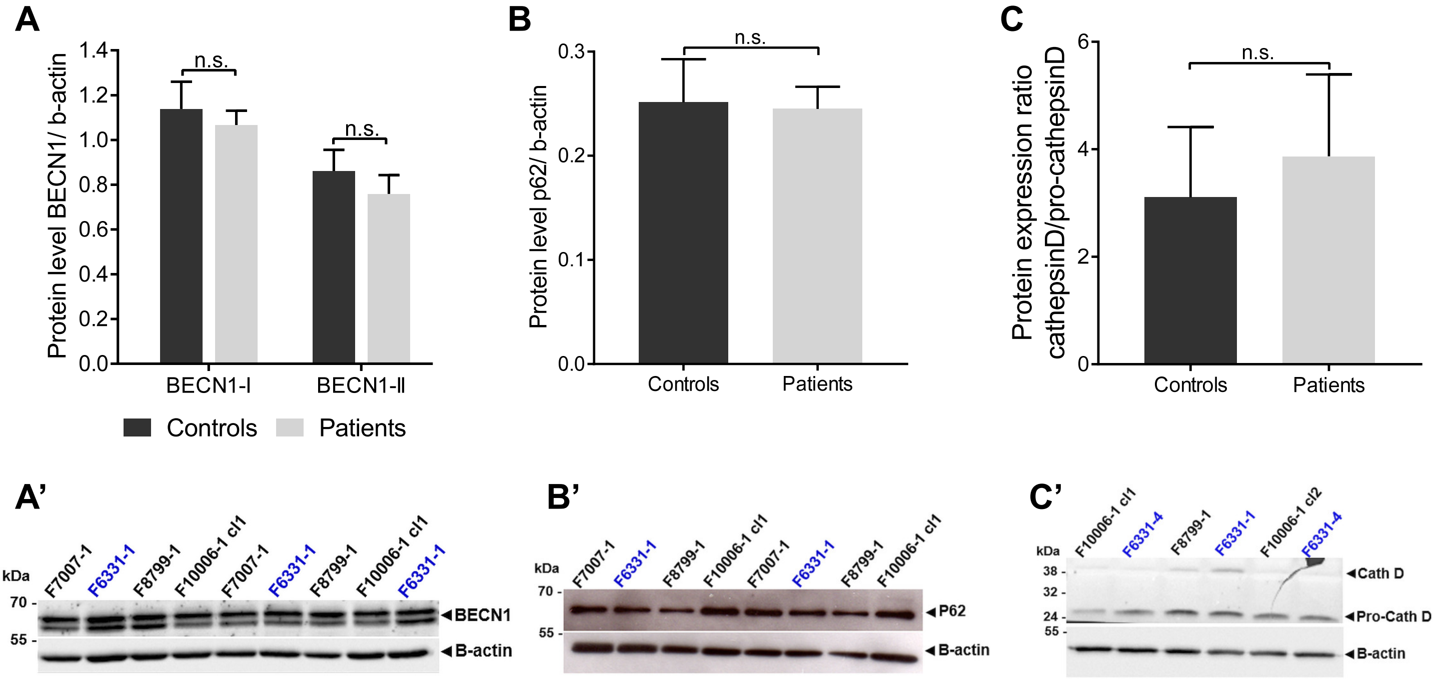

Supplementary Figure 7: Western blot for autophago-lysosomal markers in iNPC cultured in the absence of EGF/FGF-2 for 24 hours.

A, A') Blots of BECN1, showing two bands (band I and II);

B, B') Blots of p62/SQSTM1;

C, C') Blots of cathepsin D, showing cathepsin and pro-cathepsin.

β-actin was used as a loading control. The molecular mass is indicated in kDa.

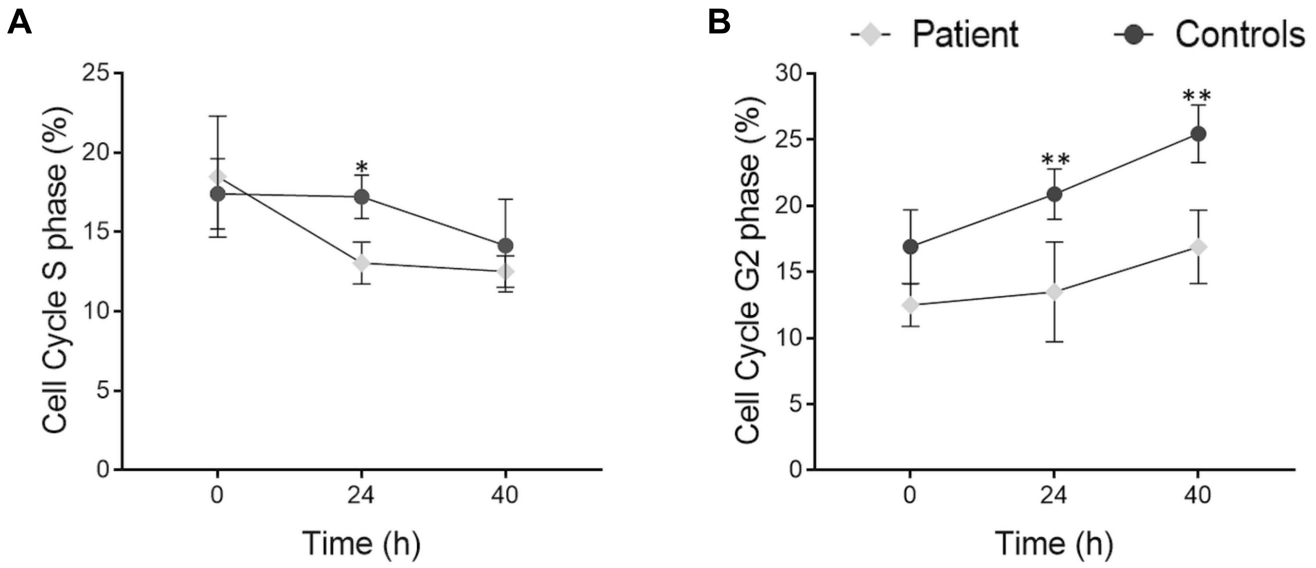

Supplementary Figure 8: Analysis of cell cycle phases in iNPC.

Graphs show the percentage of cells in S (A) and G2/M (B) phases of the cell cycle.

The analyses were performed at 3 time points (0, 24 and 48 hours). *p-value < 0.05; **p-value < 0.01. Cell cycle experiments were replicated twice. Data are shown as percentages of cells in patient's and controls' biological replicates.

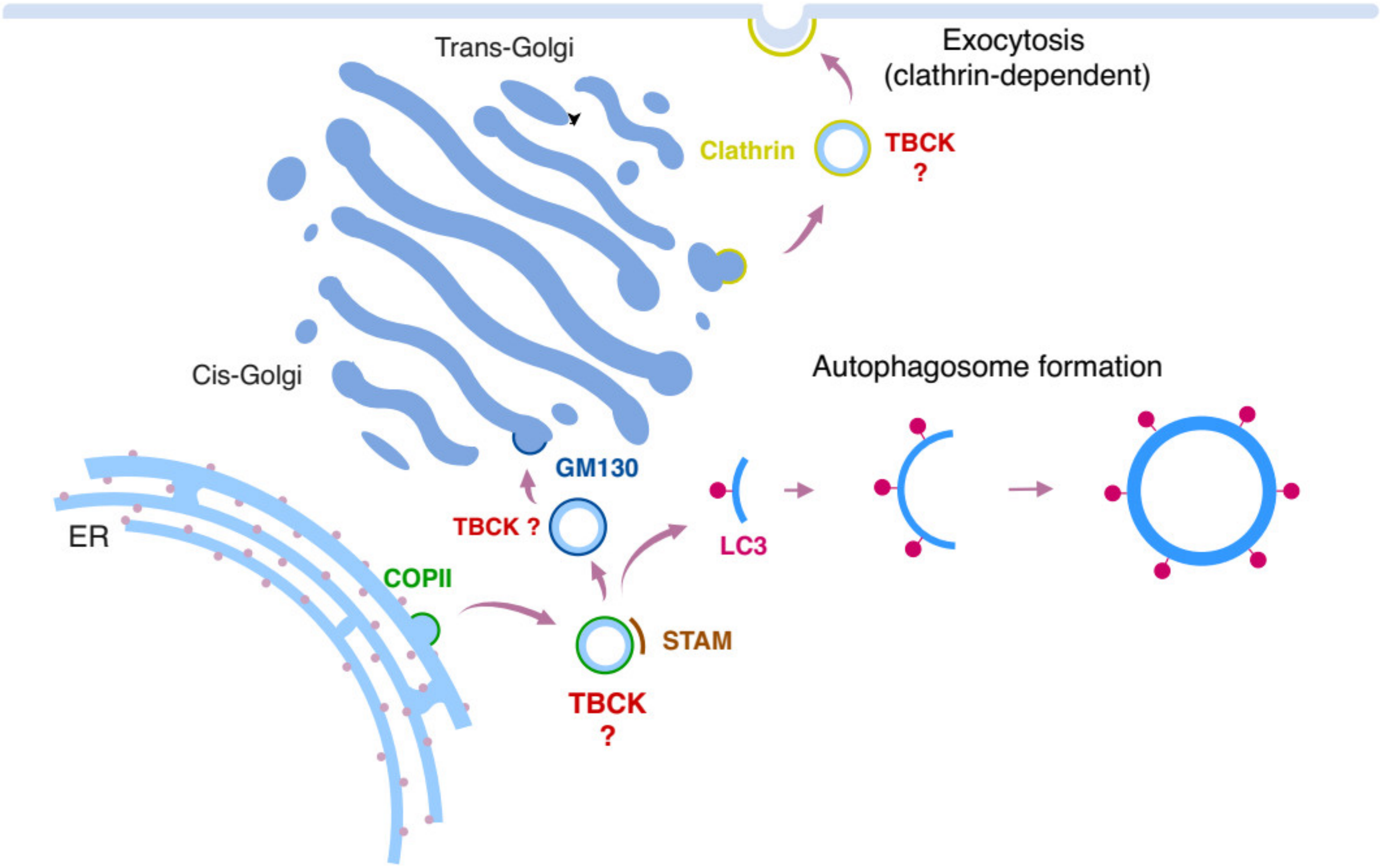

Supplementary Figure 9: Model of *TBCK* mechanism of action and IHPRF3 pathogenesis.
